# Posttranslational Regulation of Mammalian Sulfur Amino Acid Metabolism

**DOI:** 10.3390/ijms26062488

**Published:** 2025-03-11

**Authors:** María Ángeles Pajares

**Affiliations:** Department of Molecular and Cellular Biosciences, Centro de Investigaciones Biológicas Margarita Salas (CSIC), Ramiro de Maeztu 9, 28040 Madrid, Spain; mapajares@cib.csic.es

**Keywords:** methionine cycle, S-adenosylmethionine, homocysteine, posttranslational modification, transsulfuration, redox regulation, oligomerization state, subcellular localization, phosphorylation, acetylation

## Abstract

Metabolism of the mammalian proteinogenic sulfur amino acids methionine and cysteine includes the methionine cycle and reverse transsulfuration pathway, establishing many connections with other important metabolic routes. The main source of these amino acids is the diet, which also provides B vitamins required as cofactors for several enzymes of the metabolism of these amino acids. While methionine is considered an essential amino acid, cysteine can be produced from methionine in a series of reactions that also generate homocysteine, a non-proteinogenic amino acid linking reverse transsulfuration with the methionine and folate cycles. These pathways produce key metabolites that participate in synthesizing a large variety of compounds and important regulatory processes (e.g., epigenetic methylations). The impairment of sulfur amino acid metabolism manifests in many pathological processes, mostly correlated with oxidative stress and alterations in glutathione levels that also depend on this part of the cellular metabolism. This review analyzes the current knowledge on the posttranslational regulation of mammalian sulfur amino acid metabolism, highlighting the large number of modification sites reported through high-throughput studies and the surprisingly limited knowledge of their functional impact.

## 1. Introduction

Posttranslational modifications (PTMs) in proteins regulate their function, oligomerization, protein–protein interactions, and/or localization. PTMs can be reversible or irreversible, their introduction takes place either enzymatically or non-enzymatically, and their removal can be carried out by specific enzymes. Some PTMs mark the modified proteins for degradation; hence, their elimination concomitantly occurs with proteolysis of the target protein. There are hundreds of PTMs known to date [1,2], several of which may occur at the same position in the protein sequence (e.g., Lys residues can be methylated, acetylated, etc.), exerting diverse effects on the behavior of their target protein as well as allowing crosstalk between several such PTMs. In many cases, assignment of PTMs is difficult due to a lack of appropriate tools for enrichment, the compatibility of the identified sites with several related modifications (e.g., ubiquitylation and neddylation), their reversibility, or their conversion into more stable or irreversible PTMs. Nevertheless, there have been many efforts devoted to mapping a diversity of PTMs using high-throughput (HTP) methods, the results of which are available in databases such as PhosphoSitePlus [3,4]. Altogether, the combination or crosstalk between PTMs generates an array of proteoforms for each target that governs the particular behavior of a pathway under physiological or pathological conditions [5].

**Figure 1:**
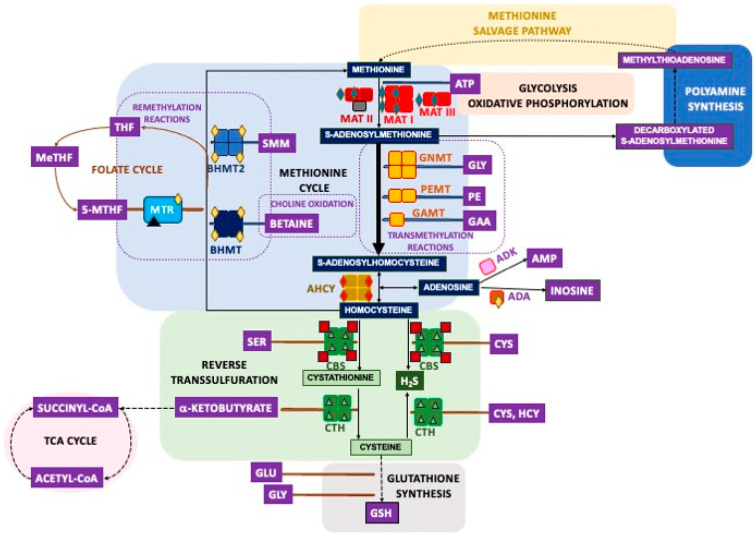
Mammalian sulfur amino acid metabolism and its main connections. The center of the figure depicts the methionine cycle and reverse transsulfuration pathways, while their links to additional routes are shown on the left side (the folate and TCA cycles), below (glutathione synthesis), at the center (choline oxidation), and on the right side (glycolysis and oxidative phosphorylation, methionine salvage pathway and polyamine synthesis). Enzymes are shown at their known association states: tetramers (MAT I, GNMT, AHCY, CBS, CTH, BHMT, and BHMT2), heterotrimers (MAT II), dimers (MAT III and PEMT), and monomers (GAMT, MTR, ADK, and ADA). Main cofactors and required ions are indicated as follows: zinc (yellow diamonds), copper (red diamonds), magnesium and potassium (blue diamonds), vitamin B_6_ (green triangles), vitamin B_12_ (black triangles), and heme group (red squares). Metabolites are shown in squares: dark blue (methionine cycle), green (reverse transsulfuration), and others (purple). Reversible reactions are indicated with double-headed arrows, discontinuous arrows indicate more than one reaction. Abbreviations: ADA, adenosine deaminase; ADK, adenosine kinase; AHCY, S-adenosylhomocysteine hydrolase; AMP, adenosine monophosphate; BHMT, betaine homocysteine S-methyltransferase; CBS, cystathionine β-synthase; CTH, cystathionine γ-lyase; GAA, guanidinoacetate; GAMT, guanidinoacetate N-methyltransferase; GNMT, glycine N-methyltransferase; GSH, glutathione reduced form; HCY, homocysteine; H_2_S, dihydrogen sulfide; MAT, methionine adenosyltransferase; MeTHF, methylene tetrahydrofolate; 5-MTHF, 5-methyltetrahydrofolate; MTR, methionine synthase; PE, phosphatidylethanolamine; PEMT, phosphatidylethanolamine N-methyltransferase; SMM, S-methylmethionine; THF, tetrahydrofolate.

This large variety of PTMs also requires a diversity of donors for protein modification, inluding metabolites of intermediary metabolism (e.g., acetyl-CoA) and gasotransmitters (e.g., nitric oxide) as well as drug metabolites and antibiotics (e.g., NAPQI or ampicillin), among others. Introduction of PTMs depends on the availability of donors, a fact that is not limiting for ATP-dependent PTMs, since intracellular concentrations of ATP can reach 1–10 mM with a half-life of nearly 60 min [6]. However, this is not always the case, as the intracellular concentrations of other donors may lie within the low micromolar or nanomolar range and be subject to oscillations that can be diet-dependent. Sulfur amino acid metabolism is of special interest in the context of PTMs as a provider of several donors used in modifications affecting a wide array of processes (reviewed in [7]) (Figure 1). The first of these compounds is S-adenosylmethionine (AdoMet), the main methyl donor for transmethylation reactions (e.g., epigenetic methylations), with intracellular concentrations between 10–90 μM and a 5 min half-life [8]. The second is homocysteine (Hcy), which is used for protein S- and N-homocysteinylation. The third is dihydrogen sulfide (H_2_S), the gasotransmitter utilized for S-sulfhydration (also named persulfidation). Finally, this pathway supplies cysteine for synthesis of glutathione, the metabolite required for protein glutathionylation, and also for the control of oxidative stress and drug detoxification, the latter rendering reactive metabolites serving for additional protein modifications.

Changes in the levels of key metabolites of sulfur amino acid metabolism are known to take place under physiological or pathological conditions; hence, these changes need to be finely tuned to avoid cell damage. In fact, dysregulation of this pathway has been detected in a variety of pathological contexts, including major liver diseases (e.g., cirrhosis and hepatocellular carcinoma (HCC)) as well as sensory impairments (e.g., hearing loss) and hereditary conditions (e.g., homocystinuria) (reviewed in [7,9]). Moreover, hyperhomocysteinemia (HHcy) has been associated with a higher risk of cardiovascular disease (reviewed in [10]). Therefore, detailed knowledge of its regulation becomes of paramount importance. This review compiles the current information on the regulation of mammalian sulfur amino acid metabolism by PTMs and the large number of modification sites identified in HTP studies, for which scarce functional information is available. The impact of PTMs with well-established roles within this pathway through their control of activity, interactions, and/or subcellular localization is considered, and putative hotspots for modification are deduced from the available data. Special attention is paid to the most abundant enzymes in the liver; studies carried out with tumoral cells or peripheral tissues may lack information about some of these proteins, which may exhibit very low expression in non-hepatic or pathological samples.

## 2. Overview of the Mammalian Sulfur Amino Acid Metabolism

Sulfur-containing amino acids comprise proteinogenic methionine and cysteine and non-proteinogenic Hcy. Methionine is considered an essential amino acid for mammals and, in this context, its levels depend on dietary intake [11]. Nevertheless, in a tumoral context, a small percentage of this amino acid (<2%) can be obtained from serine [12,13]. Methionine is the first amino acid incorporated during protein synthesis and the source of AdoMet, the main cellular methyl donor produced in the methionine cycle (Figure 1). Hcy connects this latter pathway with reverse transsulfuration, enabling maintenance of cysteine levels when required, as well as the synthesis of H_2_S. The methionine cycle is also linked to the folate cycle and choline metabolism through remethylation reactions, which permit methionine recovery using additional methyl donors generated in those pathways, 5-methyltetrahydrofolate and betaine, respectively [7,9]. All these routes are highly dependent on the intake of macronutrients (e.g., methionine and choline) and micronutrients (e.g., B vitamins), the latter acting as cofactors for some of the enzymes involved. In spite of the importance of sulfur amino acid metabolism and its connecting pathways for cell function, their study has been mostly restricted to the liver, which processes nearly 48% of ingested methionine [14,15]. This focus on the liver has led to a lack of in-depth knowledge of these pathways in other tissues, especially peripheral tissues, and to uncertain extrapolation of their performance and regulation. Additionally, studies with cell lines have relied on the use of tumoral cells, which have an elevated rate of transmethylation and exhibit the Hoffmann effect. This effect is described as their high dependence on methionine that cannot be obtained from Hcy [16,17]. Recent decades have seen advances in understanding regarding the structure of many sulfur amino acid metabolism enzymes, their expression, subcellular localization, and posttranslational regulation, this final aspect being addressed especially through HTP methods.

The methionine cycle is essentially a cytosolic pathway, although recent data have identified distinct enzymes in additional subcellular compartments, such as the nucleus [18,19,20,21,22,23,24] or the mitochondria [25]. The first reaction in this route catalyzed by methionine adenosyltransferases (MATs) produces 6–8 g AdoMet daily from methionine and ATP. Mammals have three MAT genes, *MAT1A* and *MAT2A* encoding the catalytic subunits MATα1 and MATα2, respectively, and *MAT2B* codifying for the regulatory subunit MATβ. These genes are considered essential for life and as such were included in the genome of the first synthetic organism generated [26,27]. Expression of MAT genes differs among tissues, developmental stages, and pathological states. In fact, *MAT1A* has long been considered a liver-specific gene, due to its low expression in other tissues [18], while *MAT2A* and *MAT2B* are classified as being of extrahepatic, fetal, or pathological expression, since their detection is high in those tissues and conditions. Homodimers of catalytic subunits are the minimum association state allowing AdoMet synthesis, as shown in the available crystal structures, where active sites (two/dimer) are located at the interface between the two monomers, both of which contribute residues for catalysis and substrate binding [28,29,30]. Classical studies isolated three cytosolic isoenzymes from tissues and named them according to their behavior on phenyl Sepharose beads, as follows: MAT I (MATα1 homotetramer); MAT III (MATα1 homodimer); and MAT II (heterotrimer composed of a MATα2 homodimer and one MATβ subunit). Nevertheless, recombinant MATα2 homotetramers have been obtained and characterized [31]. MAT oligomers differ in their affinities for methionine (MAT II > MAT I > MAT III) and their V_max_ (MAT II < MAT I < MAT III), as well as in their feedback inhibition by AdoMet (reviewed in [32,33]). Moreover, the methionine affinity and V_max_ of MAT II are modified by NADP^+^ binding to the MATβ subunit [31]. Hence, the intracellular AdoMet level depends on which isoenzymes are present and, therefore, the pathological importance of the changes in expression patterns found in many hepatic pathologies. Recent studies have also shown that differences in expression levels may correlate with variations in subcellular distribution of the proteins. In fact, low *MAT1A* expression in extrahepatic tissues and its decrease in liver pathology correlated with increased nuclear localization of the protein [18,34], which may be key to maintaining nuclear AdoMet levels for essential cellular events. Both MATα1 and MATα2 oligomers interacted with the low-Mr protein p53 and DNA damage-regulated gene 1 (PDRG1), which inhibited their catalytic activities in vitro [35]. However, despite the high degree of identity between MATα1 and MATα2 sequences, the areas of PDRG1 involved in each interaction seem to have been different and MATβ was displaced from its binding site on the MATα2 dimer [35]. Moreover, immunoprecipitation experiments restricted the occurrence of MATα1–PDRG1 interaction to the nucleus [35]. The importance of PDRG1 relies on its presence in a variety of macromolecular complexes involved in splicing and chromatin remodeling, among other purposes, where its role, linked or not to the control of MATα binding and/or activity, remains unknown (reviewed in [36]). Additionally, the existence of alternative splicing forms, two for MATα2 [37] and four for MATβ [38], has been described, although their characterization is very limited.

The huge variety of AdoMet-dependent methylation reactions includes protein, DNA and RNA methylations, as well as the synthesis of small compounds such as phospholipids or neurotransmitters [39]. The methyltransferases involved use nearly 95% of the AdoMet produced and render its demethylated form, S-adenosylhomocysteine (AdoHcy). The remaining AdoMet is diverted to polyamine synthesis after its decarboxylation [40] or used in minor processes such as those catalyzed by SAM radical proteins that require 5’-deoxyadenosyl radicals [41,42]. Methylation reactions do not take place only in the cytosol; thus, there is a need for specific carriers to allow exchange of AdoMet and AdoHcy from compartments such as the mitochondria [43]. Altogether, these reactions link the methionine cycle with a vast array of pathways, whose contribution and importance depend on specific functions of each cell or tissue, the liver being the main consumer of AdoMet methyl groups. For example, glycine N-methyltransferase (GNMT), phosphatidylethanolamine N-methyltransferase (PEMT), and guanidinoacetate N-methyltransferase (GAMT) are highly expressed in the liver and known to be the main consumers of hepatic AdoMet for the synthesis of sarcosine, phosphatidylcholine, and creatine, respectively. In contrast, the low or lack of expression of *GNMT* and *PEMT* in extrahepatic tissues may convert their contribution to residual in that context. Again, the structures of most methyltransferases are becoming available, as is the case of cytosolic GNMT homotetramers and GAMT monomers, whereas that of the PEMT homodimers remains elusive as for many membrane proteins of the endoplasmic reticulum. Additionally, splicing forms appear listed in the NCBI-SNP database, and the PEMT case was reported by Shields et al. [44].

AdoHcy elimination by AdoHcy hydrolase (AHCY) is crucial for cell function as it inhibits many methyltransferases, the activity of which depends on the existence of a suitable intracellular AdoMet/AdoHcy ratio, which may vary for each cell type. Homotetrameric AHCY catalyzes a reversible reaction producing Hcy and adenosine, products that need to be rapidly removed to avoid AdoHcy resynthesis. Adenosine is then used by monomeric enzymes, adenosine deaminase (ADA) or adenosine kinase (ADK), the latter being the preferred reaction in adult hepatocytes. The high affinity of ADA favors removal and leads to inosine and, subsequently, hypoxanthine, whereas the reversible reaction catalyzed by ADK renders AMP for ATP recycling. On the other hand, Hcy can be removed to the extracellular space, remethylated for methionine recovery by monomeric methionine synthase (MTR) and homotetrameric betaine homocysteine S-methyltransferases (BHMT and BHMT2), or catabolized to cystathionine by homotetrameric cystathionine β-synthase (CBS), subsequently entering reverse transsulfuration. It is noteworthy that an excess of Hcy can be used for the posttranslational modification of proteins on cysteines (S-homocysteinylation) or lysines (N-homocysteinylation) [10]. Hcy remethylation enzymes use diverse methyl donors; MTR consumes 5-methyltetrahydrofolate produced in the folate cycle, whereas BHMT utilizes betaine obtained from the diet or through choline oxidation and BHMT2 employs dietary S-methylmethionine. Moreover, the three enzymes require Zn^2+^, and MTR also needs cobalamin (vitamin B_12_) as a cofactor. The ubiquitous *MTR* expression pattern suggests an essential crosstalk with the folate cycle in every mammalian cell and tissue, whereas connection with choline metabolism may be less relevant, except in the liver, where *BHMT* genes are highly expressed in hepatocytes. Nevertheless, we should not forget that *BHMT* expression also occurs to a limited extent in certain extrahepatic tissues and cell types, correlating with its nuclear localization [19], in turn, indicating a putative link with key nuclear events. Kinetic expression and structural information for many of these oligomeric enzymes is now available, although not yet for MTR, for which only structures of certain domains are known. Splicing forms for most of these enzymes have been reported, although in some cases their detection seems restricted to a single tissue, as is the case for a MTR splicing form found in brain tissue [45].

Reverse transsulfuration is mainly a cytosolic pathway, although some enzymes have been also detected in the cell nucleus [23]. The first reaction involved is catalyzed by CBS, which condenses Hcy and serine to generate cystathionine, but this enzyme is promiscuous and thus can use additional substrates in reactions that produce H_2_S. An alternative splicing form of the enzyme has been described [46], as well as its nuclear localization [23]. CBS is a homotetramer that depends on pyridoxal phosphate (vitamin B_6_), has a heme cofactor, and exhibits low affinity for Hcy. Thus, remethylation is favored as long as Hcy levels remain low, whereas CBS activity is stimulated by increases in AdoMet levels. The crystal structure shows that the monomer is organized into three domains: N-terminal, central, and C-terminal, which bind heme, B_6_, and AdoMet, respectively. In the AdoMet-free conformation, the C-terminal domain blocks access to the active site in the neighboring subunit of the tetramer. Mutations in CBS are the main origin of autosomal recessive homocystinuria (OMIM #236200) [47]. Cystathionine is catabolized to produce cysteine by homotetrameric cystathionine γ-lyase (CTH), another promiscuous B_6_-dependent enzyme that synthesizes H_2_S from cysteine and for which splicing forms are also listed in the NCBI-SNP database. B_6_ binds to the N-terminal domain of the subunit, while the second domain comprising the rest of the protein contains a tyrosine residue involved in propargylglycine inhibition that stacks against the pyridinium ring of the vitamin. In several cell types, there is a lack of or minimal expression of CBS and 3-mercaptopyruvate sulfurtransferase, the other enzymes able to synthesize H_2_S, and hence, CTH becomes mainly responsible for the production of this gasotransmitter under physiological substrate concentrations. Thus, the flux through reverse transsulfuration is governed by protein expression levels. The abundance of cofactors and substrates as has been summarized in several reviews [7,48,49,50].

## 3. Methods and Sample Types Used for the Identification of Posttranslational Modifications in the Main Enzymes of Sulfur Amino Acid Metabolism

High-throughput (HTP) methods have enabled the identification of an enormous number of PTM sites in proteins. For this purpose, label-free control and treated cell lines (Table 1) or, less frequently, tissues (Table 2), as well as SILAC labeled samples have been used. Lysates and, less frequently, subcellular fractions have been obtained in the presence of appropriate inhibitors and digested using mainly trypsin, either alone or combined with e.g., Lys-C. The modified peptides have been captured for enrichment using a variety of methods selected according to the characteristics of the PTM under study (Table 1 and Table 2). These include the utilization of specific antibodies (e.g., anti-acetyl-Lys), ion exchange chromatographies (e.g., strong cation exchange (SCX)), and/or immobilized metal affinity resins (e.g., IMAC) before identification using LC-MS/MS. In some instances, results from two cell lines or several tissues have been reported and compared, thus allowing the classification of the modification sites as cell/tissue-specific or not. The same is true for some treatments, with the classification of the PTM site as responsive or non-responsive to the agent or condition of interest.

HTP studies reporting PTM sites in enzymes of the mammalian sulfur amino acid metabolism have focused on wide-impact modifications such as ubiquitylation, sumoylation, acetylation, N-glycosylation, methylation, and phosphorylation, although others of apparently less importance such as succinylation are also being uncovered. The results obtained provide a map of PTMs for each protein of interest, as can be found in databases such as PhosphoSitePlus^®^ (https://www.phosphosite.org). However, the functional importance of the specific sites remains unexplored in most cases, except for a few that have been specifically analyzed. This highlights the fact that the mapping of PTM sites on a specific protein needs to be followed by a thoughtful analysis of their functional consequences, work that requires specific methodologies. This manuscript does not reproduce the PTM maps that can be found in databases, but it instead summarizes and discusses the known and putative functional impact of modifications in the enzymes of interest. For clarity, throughout the text of the current paper, human residues are mentioned only by their one-letter code and sequence number (in some manuscripts, numbering does not take into account removal of the initial methionine), whereas those identified in rat and mouse samples are indicated in superscript with R or M, respectively.

## 4. Regulation by Phosphorylation

**Figure 2:**
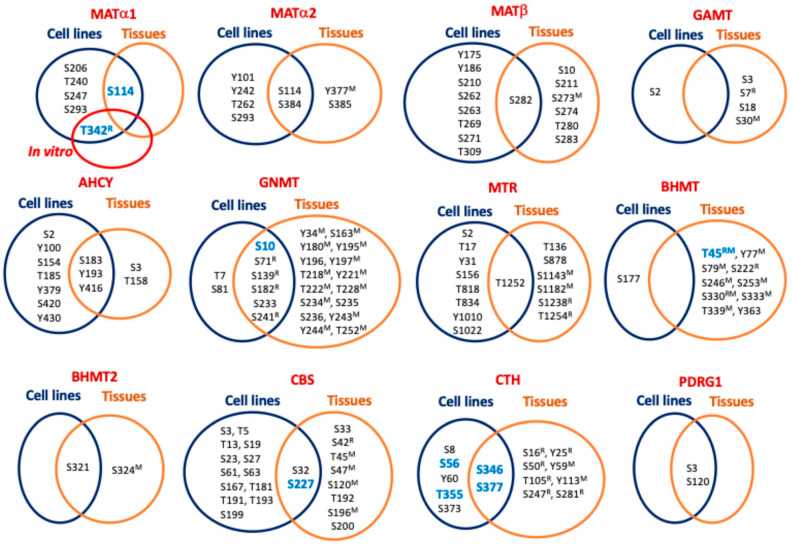
Phosphorylation sites identified in enzymes of mammalian sulfur amino acid metabolism. Modification sites found in cell lines (blue circles), tissue samples (orange circles) and in vitro (red circles) are depicted. Intersections between circles contain the phosphorylation sites identified in several types of samples. Superscripts are used to label phosphorylation sites found in rat (R) and mouse (M) samples. Information about PDRG1, a MATα interaction target, is also included. Modification sites for which functional information is available appear highlighted in bold blue font. Residue numbering reported in the literature may not coincide with the sequence position due to excision of the initial Met.

Phosphorylation is the most studied PTM and its enzymatic introduction was deciphered in the 1950s [124]. The catalogue of kinases and phosphatases has grown ever since, together with the interest in their signaling pathways in physiological and pathological contexts (reviewed in [125]). HTP studies have identified phosphorylation sites in all the main enzymes of sulfur amino acid metabolism, using cell lines and tissue samples (Table 3), although in many cases, the coincidence is scarce (Figure 2). These studies have been limited to the identification of main target residues (Ser, Thr, and Tyr) and, to the best of the author’s current knowledge, no report on other putative phosphorylated residues has been published. Meanwhile, experiments using purified or recombinant enzymes, as well as mutants on the specific residues that mimic or suppress the modification, provided the identification of additional phosphosites and some information about their functional impact.

### 4.1. Phosphorylation of Methionine Adenosyltransferases

**Figure 3:**
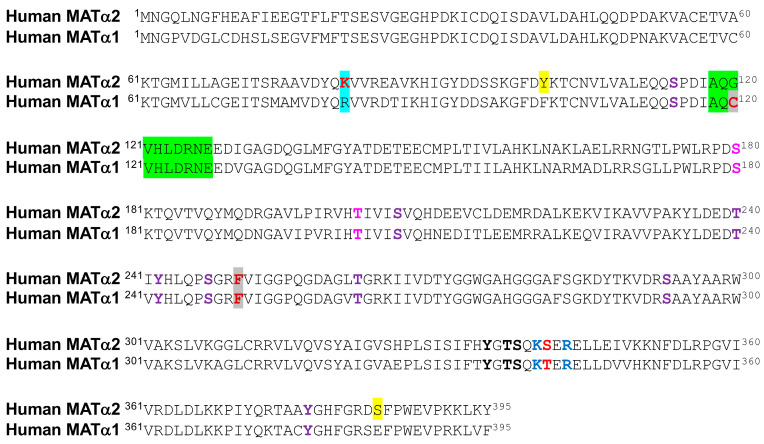
Comparison of human methionine adenosyltransferase sequences using BLAST (https://blast.ncbi.nlm.nih.gov/). Sequences of the human catalytic subunits MATα1 and MATα2 are shown and the residues of interest are highlighted as follows: red, MATα1 T341 equivalent to the PKC phosphosite found in rat and the equivalent S341 in MATα2; blue, conserved residues of the conformational location signal identified in rat MATα1 (K340 and R343) located near T341; red on grey background, MATα1 residues C120 at the loop of access to the active site and F250 involved in methionine binding; black, MATα2 phosphosites Y335, T337, and S338 preserved in both catalytic subunits; purple, S114, S206, T240, Y242, S247, T262, S293, and Y377 phosphosites identified in high-throughput studies and conserved in both MATα1 and MATα2; yellow background, Y101 and S284 non-conserved MATα2 phosphosites identified in high-throughput studies; green background, active site loop A118-E128; fuchsia, AKT2 phosphorylation sites in MATα1 (S180 and T202) conserved in MATα2; red on blue background, MATα2 acetylation site (K81), position occupied by R81 in MATα1.

Interestingly, phosphorylation of MATα1 oligomers seems to be related to the regulation of their association state and, directly or indirectly, with the subcellular localization of the protein. Initial studies with purified MAT I and MAT III showed their PKC phosphorylation on T342^R^ [126], modification of the equivalent human T341 being later reported in Jurkat cells (PhosphoSitePlus curated record 3360104). This phosphorylation diminished monomer–monomer interactions, in turn altering enzyme activity [126]. The location of T342^R^ in the C-terminal segment at the interface between dimer subunits and within an 11–14 Å distance of residues in the opposite monomer may explain these effects. Moreover, T342^R^ is flanked by K340^R^, K341^R^, and R344^R^, which are part of the conformational signal regulating the nucleocytoplasmic distribution of MATα1 [18]. Mutation of these three lysine residues to alanine decreases MATα1 nuclear content, therefore suggesting that phosphate in this microenvironment may also alter subcellular distribution. Of note, equivalent residues to K341^R^ and R344^R^ are conserved in human MATα1 and MATα2, whereas in the latter, T341 is substituted with Ser (Figure 3).

Additionally, tiny amounts of MATα1 have also been detected in mitochondria [25], where its interaction with enzymes of the TCA cycle, oxidative phosphorylation, and β-oxidation of fatty acids was uncovered [127]. Impairment of mitochondrial MATα1 localization was observed in mouse and human models of alcohol liver disease, concomitantly with its phosphorylation on S114 by casein kinase 2. This phosphorylation favored cytosolic MATα1 interaction with peptidyl-prolyl cis–trans isomerase PIN1 but reduced its half-life [127]. In parallel, as a consequence of decreased mitochondrial MATα1 levels, interactions with mitochondrial enzymes and levels of protein lysine methylation were reduced with unknown functional consequences. The impact of S114 phosphorylation on MAT I/III activity was not analyzed in that work, although that of the S114A mutant decreased compared with the wild-type protein [127]. S114 is exposed at the protein surface in the segment preceding the loop of access to the active site (A118-E128) [28], a position in which substitution or modification could alter the movement of this loop and putatively exert some impact on substrate accessibility for catalysis. It is noteworthy that S114 is conserved in MATα2 (Figure 3), and HTP studies have identified its phosphorylation in both MATα1 and MATα2 (Figure 2) [54,69,74,75,96,114,120,121].

Increased phospho-MATα1 levels were also detected in human HCC and mouse cholangiocarcinoma samples together with MATα1-YWHAZ coimmunoprecipitation, and the interaction was confirmed with recombinant proteins [128]. Analysis of the MATα1 sequence identified two canonical YWHAZ binding motifs with potential AKT2 phosphosites. Mutants on these positions, S180A and T202A, or *YWHAZ* silencing induced MATα1 nuclear localization, while diminishing its interaction with YWHAZ. Crystal structures suggest that S180 is not easily accessible with its side chain hidden inside the MATα1 monomer and next to F251^R^ and the methionine binding site. Conversely, modification of T202 is feasible as its side chain is oriented towards the surface of each monomer at the limit of the interface between dimer subunits. This location suggests a putative role for T202 modification in MATα1 dissociation and its subsequent entrance into the nucleus, where the monomer is the main form encountered [18]. Nonetheless, the precise phosphosite involved in these events has not been unequivocally identified, nor have these modified residues been found in HTP studies (Figure 2). Interestingly, both S180 and T202 are conserved in MATα2 (Figure 3), but their phosphorylation has not been reported.

Purifications of MAT II showed doublet bands in MATα2, suggestive of the presence of PTMs in the protein [129,130]. Subsequent studies reported phosphorylation of both MATα2 and MATβ subunits and their enhanced stability in human stellate cell trans-differentiation [131]. Both subunits were substrates for MEK phosphorylation, but ERK1/2 and B-Raf targeted only MATβ. Several phosphopeptides were identified for each subunit, and double and triple mutants on some of these phosphosites were analyzed, showing impaired interaction between regulatory (Y259F/T257V) and catalytic mutant subunits (Y371F/T374F and Y335F/T337V/S338A) [131]. However, a close look into the available structures suggests limited kinase accessibility to Y371 and T374, located inside the MATα2 dimer (PDB 2P02). In contrast, Y335, T337, and S338 are situated at or close to the interface between MATα2 subunits; in particular, Y335 is located inside a cleft at this interface. Interestingly, these three residues are conserved between human MATα2 and MATα1 (Figure 3). Regarding the regulatory subunit, Y259 is located at the external surface (PDB 2YDY; [132]), and its phosphorylation may alter hetero-oligomerization by changing the orientation of an α-helix that extends towards the binding interface with the MATα2 dimer. Remarkably, HTP studies included in PhosphoSitePlus did not identify any of these MATα2 and MATβ phosphosites. Studies that evaluated phosphorylation during the cell cycle in HeLa–Fucci cells or in mycobacterial infection of RAW 264.7 macrophages described increased nuclear MATα2 content during S + G2 phases [133] or detected MATβ in a band recognized with anti-phosphoTyr [134], respectively, without further identification of the phosphosites involved. Finally, although HTP studies have identified some phosphosites in PDRG1 (Figure 2), the small protein that inhibits MAT activity, no functional information is available to date.

### 4.2. Phosphorylation of Main Hepatic Methyltransferases

Regarding methyltransferases, the large number of these enzymes makes it necessary to focus on a few. For this purpose, we have selected the three main hepatic consumers of AdoMet, which are GAMT, GNMT, and PEMT. HTP studies have reported phosphorylation of GAMT and GNMT (Table 3) but have generally lacked information about PEMT modification, since membrane proteins have not been extensively analyzed with these methods. Nevertheless, specific studies on glucagon- or cAMP-stimulated hepatocytes suggested putative PEMT phosphorylation by PKA leading to increased activity [135]. In vitro experiments with purified PEMT demonstrated its PKA and PKC modification, both enhancing activity [136,137,138]. In contrast, no such specific studies on GAMT phosphorylation have been published, and the functional relevance of the phosphosites identified by HTP hence remains unknown. Moreover, S30^M^ is not conserved in human GAMT and there is no overlap between phosphosites in cell lines and tissue samples (Figure 2).

On the other hand, GNMT phosphorylation has been identified in purified rat liver and recombinant enzymes and in immunoprecipitates from hepatocytes and liver samples. Several phosphosites (S71^R^, S182^R^ and S241^R^) were common for all sample types (Figure 2), whereas S9^R^ and S139^R^ modifications were detected only in the liver and recombinant enzymes, respectively, and both were identified in hepatocytes [139]. Among these residues, S9^R^ and S182^R^ of opposite monomers are placed within a short distance at the surface of the rat GNMT homotetramer crystal structure (e.g., ~13Å between S182^R^ of monomers A and B in the 3THR PDB structure), a fact that led the authors to suggest a putative destabilizing effect of the modification. Previous work had already described enhanced GNMT activity by PKA phosphorylation [140], while new in vitro assays showed S9^R^ as the target for this kinase [139]. Moreover, 5-methyltetrahydrofolate binding inhibited GNMT and, in this cofactor-bound state, PKA phosphorylation was precluded [140]. Additionally, studies with FLAG-GNMT transfected cells showed T7 and S9 phosphorylation in controls with DMSO and cells treated with benzo(A)pyrene, respectively [141]. Modification on S9 correlated with nuclear accumulation of GNMT, which was precluded in the S9A mutant [141]. Additionally, experiments including mutants and distinct kinase inhibitors further suggest that benzo(A)pyrene-induced S9 phosphorylation may depend on PKC and JNK [141]. Thus, like MATs, phosphorylation is involved in the regulation of subcellular localization.

### 4.3. Phosphorylation of S-Adenosylhomocysteine Hydrolase and Remethylation Enzymes

Data about phosphorylation of enzymes involved in the production and remethylation of Hcy are mostly limited to HTP studies (Table 3). Interestingly, phosphorylation of active-site-conserved positions S203 and S236 of plant AHCY1 has been reported [142]. However, modification of only S154, equivalent to plant S203, has been found in AHCY using HTP studies and mammalian cell lines (Table 3). Overlap between phosphosites identified in cell lines and tissues is restricted to S183, Y193, and Y416 of AHCY and only T1252 of MTR, and it is lacking in BHMT (Figure 2). Nevertheless, specific studies in hepatocytes have shown T45 phosphorylation in BHMT and uncovered its interaction with ribosomal modification protein rimK-like family member A (RIMKLA) [143]. T45 modification correlated with increased BHMT activity in RIMKLA-overexpressing cells and decreased Hcy levels, leading in turn to reduced FASn and CD36 protein levels, diminished lipid deposition and uptake in hepatocytes [143]. All these effects were impaired by expression of a T54A BHMT mutation. The same study also analyzed several models and samples of liver disease (e.g., non-alcoholic fatty liver disease), showing decreased RIMKLA expression with consequent impact on BHMT phosphorylation and lipid metabolism [143]. Structurally, T45^R^ is exposed at the protein surface, with its hydroxyl group placed at ~13Å of K402^R^ on the C-terminal α-helix that fastens the monomers. Introduction of a phosphate group on T45^R^ may shorten this distance, allowing stronger interactions with the K402^R^ side chain and contributing to enhancing BHMT tetramer stability.

### 4.4. Phosphorylation of Reverse Transsulfuration Enzymes

Information from HTP studies of cell lines and tissues also shows minimal overlap of the phosphosites identified in CBS and CTH (Table 3). Conversely, specific studies were designed mainly to uncover the putative regulation of H_2_S production in several contexts. For example, this gasotransmitter regulates urine bladder relaxation, and its production, and that of cGMP, was enhanced by carbachol stimulation of muscarinic receptors in urothelium and urothelial cells [144]. Experiments combining carbachol stimulation with CBS or CTH inhibitors or just the treatment of *CBS* knockout cells ascribed the increased H_2_S production solely to CBS activation due to augmented S277 phosphorylation levels [144]. This residue is a known PKG target and its role in the urothelium was further proven with the use of inhibitors of PKG and nitric oxide (NO) production. In the crystal structure, S227 is exposed at the protein surface but located in a hollow.

Specific information regarding CTH phosphosites has been obtained from endothelial cells under hypoxia [145], after stimulation of the G-protein-coupled bile receptor GPBAR1 [146,147], via 17β-estradiol induction [148], after IL-1β treatment [149], and by application of disturbed flow [150]. Conditions in which increased enzyme activity has been reported have not always resulted in increased H_2_S levels. Hypoxia led to S346 and T355 phosphorylation and enhancement of CTH activity, which was precluded or mimicked by the corresponding alanine and glutamic acid mutants, respectively. Interestingly, under hypoxia, these glutamic acid mutants increased persulfide and polysulfide intracellular levels in the presence of cysteine, cistine, or cystathionine, but not H_2_S levels [145]. Furthermore, S346 was a predicted target for AMPK and a correlation between increased kinase and phopsho-S346 levels was found both in endothelial cells under hypoxia and in mouse muscle after femoral artery ligation [145]. Either *AMPK* silencing or dorsomorphin inhibition downregulated these phosphorylation events. Dynamic simulations also showed that S346 and T355 modification may favor new electrostatic interactions within and between CTH subunits, putatively resulting in a higher stability of the tetramer and B_6_ binding [145]. Conversely, 17β-estradiol induced CTH activity and H_2_S production, an effect that was mediated by increased cGMP levels and PKG-Iβ activation and required estrogen receptor interaction with the guanylate cyclase subunit GαI [148]. Moreover, 17β-estradiol treatment enhanced CTH-PKG-Iβ interaction, leading to S56 phosphorylation [148]. In the human CTH crystal structure, the side chains of S56 in monomers B and D are exposed, facing each other with their OH groups at ~25Å. However, it is difficult to envision whether introduction of phosphate groups at those positions can affect dimer interactions and enzyme activity or substrate specificity.

Enhanced CTH phosphorylation levels induced by GPBAR1 and IL-1β stimulation and after application of disturbed flow to endothelial cells seem to rely on S377 modification. GPBAR1 stimulation increased both CTH activity and cAMP levels, which, in turn, elevated *CTH* promoter activity and expression [146]. In this context, augmented CTH phosphorylation levels positively correlated with those of phospho-AKT and CTH-AKT interaction, effects that were prevented by inhibition of the PI3K/AKT pathway [146,147]. In contrast, IL-1β stimulation increased CTH phosphorylation levels but decreased H_2_S production; this effect was mimicked by mutants in several potential phosphosites, with the S377D mutant erasing CTH activity [149]. Bibli et al. also reported similar effects in murine carotid arteries after ligation and in human atherosclerotic plaques [149], as well as differences between patients receiving statins or not [150]. Samples from patients treated with statins showed decreased CTH protein and phosphorylation levels, whereas those without treatment exhibited increased CTH expression and S377 phosphorylation but low activity [150]. Additionally, a role for S377 phosphorylation in O_2_ sensing in the carotid body was suggested by work presenting an interesting interplay between three gasotransmitters [151]. NO and CO activated guanylate cyclase to produce cGMP that subsequently triggered PKG, resulting in decreased H_2_S production by CTH. Using a combination of models, including carotid bodies and cells expressing hemoxygenase 2 (HO-2) and/or CTH under normoxia or hypoxia, as well as the CO donor CORM-2 and PKG or guanylate cyclase silencing, a correlation between increased CO production and enhanced CTH phospho-serine levels was established [151]. Given the implication of PKG, S377 was suggested as the target phosphosite and its role was confirmed with the corresponding alanine and glutamic acid mutants, the S377E mutant reducing H_2_S production in cells under normoxia [151]. Analogous experiments using NO donors for guanylate cyclase activation also decreased H_2_S levels while increasing those of phospho-serine CTH; the S377A mutant abrogated these effects [151]. Altogether, these reports rely on the use of mutants or antibodies to ascribe the observed effects to the S377 phosphosite, but its side chain does not seem accessible to kinases, according to the crystal structure of human CTH with the B_6_ cofactor.

## 5. Regulation by Ubiquitylation and Sumoylation

### 5.1. Modification by Ubiquitylation

**Figure 4:**
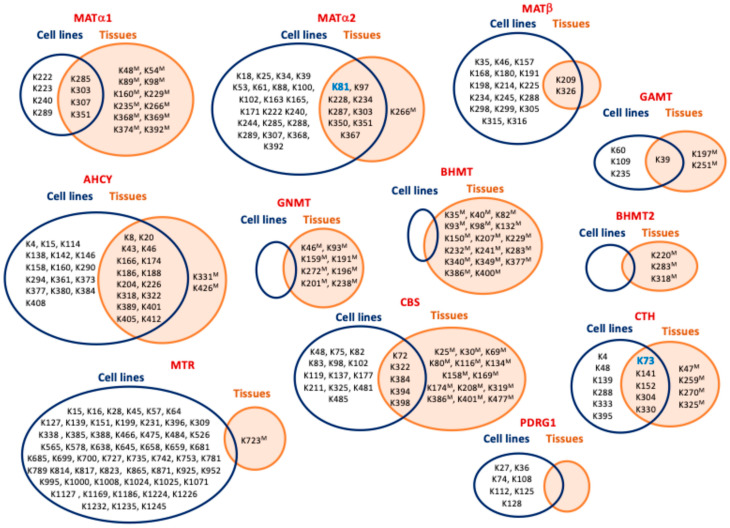
Ubiquitylation sites identified in enzymes of mammalian sulfur amino acid metabolism. Modification sites found in cell lines (blue circles) and tissue samples (orange circles) are shown. Intersections between circles contain the ubiquitylation sites identified in several types of samples. Superscript M indicates ubiquitylation sites found in mouse samples. Information about PDRG1, a MATα interaction target, is also included. Modification sites for which functional information is available appear highlighted with larger blue fonts. Residue numbering reported in the literature may not coincide with the sequence position due to excision of the initial Met, and some displacement may have occurred in the numbering of modification sites identified between mouse samples and human cell lines.

Multiple ubiquitylation sites in enzymes of sulfur amino acid metabolism have been detected in HTP studies (Table 4), although limited (MATβ and GAMT) or no overlap (GNMT, BHMT, BHMT2, MTR and PDRG1) has been found between cell lines and tissue samples in several cases (Figure 4). Additionally, studies aiming to identify the protein interaction targets of these enzymes have found several proteins associated with the ubiquitylation machinery. Cases include the MATα2 interaction targets cullin 3 (CUL3), a member of the BCR ubiquitin ligase complex serving as scaffold for protein modification and targeting for degradation [152], the E3 ligases TRIM25 [153] and UBR4 [154], the deubiquitylase VCIP135 [155], as well as the two CTH interaction targets Rad18 E3 ubiquitin ligase and its substrate scaffolding protein REV1 [156]. These reports provide more specific evidence of ubiquitylation and its crosstalk with other modifications and the involvement of certain circRNAs, but they include almost no identification of ubiquitylation sites.

In ladder tests, samples of human colorectal cancer showed inverse correlation between MATα2 and CUL3 levels and MATα2 ubiquitylation [152]. Assays in cell lines then demonstrated CUL3–MATα2 interaction and alteration of the modification levels by folate deprivation and treatment with the deacetylase inhibitor trichostatin A, the proteasomal inhibitor MG132, or *CUL3* silencing. Both trichostatin A or folate deficiency increased MATα2–CUL3 interaction, subsequently enhancing MATα2 ubiquitylation and degradation, effects abrogated in the K81R mutant [152]. An analogous study carried out in HCC and several cell lines also found similar outcomes following MATα2 modification [154]. Therefore, K81 was suggested as the ubiquitylation site. Furthermore, immunoprecipitation from folate-deprived HEK293T expressing FLAG- MATα2 identified the UBR4-E3 ligase as its interaction partner, together with MATα2 polyubiquitylation [154]. UBR4 overexpression and knockdown resulted in decreased and increased MATα2 levels, respectively [154]. UBR4 silencing blocked MATα2 degradation induced by folate deprivation but also resulted in its enhanced acetylation. Altogether, the results of these combined treatments led the authors to propose that MATα2 acetylation on K81 promotes ubiquitylation in unspecified locations and its further degradation.

Other studies have reported interesting interactions with other members of the ubiquitylation system but provided no identification of the modification sites. This was the case in the comparison between cisplatin-resistant and non-resistant bladder cancer cells that showed inverse correlation between MATα2 protein and circARHGAP10 levels and their interaction, according to the results obtained via RNA immunoprecipitation and pull-down [153]. In this context, interaction of circARHGAP10 with TRIM25, also an interaction partner of MATα2, was found. Overexpression of circARHGAP10 decreased MATα2 protein levels and stability, enhancing its ubiquitylation; this effect was prevented by MG132, whereas TRIM25 silencing increased MATα2 protein levels [153]. Yang et al. suggested circARHGAP10 as a scaffold favoring TRIM25–MATα2 interaction and ubiquitylation. Furthermore, a positive correlation between MATα2 and VCIP135 protein levels in human hepatocarcinoma was detected, whereas increased MATα2 ubiquitylation was observed upon silencing of this deubiquitylase, which is involved in p97/p47-dependent fusion of the Golgi membrane [155].

Regarding CTH, HUVEC cells treated with angiotensin II, a known inducer of endoplasmic reticulum (ER) stress in the vasculature, displayed decreased enzyme levels, enhanced ubiquitylation, and proteasomal degradation [157]. As a consequence, production of H_2_S decreased, thus reducing its ability provide protection from ER stress, whereas protection was achieved by addition of MG132. Angiotensin II was shown to increase CTH polyubiquitylation by 48-linked ubiquitin as well as superoxide levels, while these increases were prevented by N-acetylcysteine [157]. In the mouse cardiovascular system, angiotensin II also induced CTH ubiquitylation and degradation, contributing to hypertension, and these effects were precluded by N-acetylcysteine and the SIRT3 activator honokiol [158]. The effects of angiotensin II are known to involve HDAC6, and hence, several experimental models and deacetylase inhibition with tubastatin A were used to show the contribution of CTH K73 acetylation to H_2_S production [159]. Crosstalk between CTH ubiquitylation and acetylation was then uncovered; honokiol decreased ubiquitylation levels induced by angiotensin II, and acetylation followed the opposite pattern [158]. Proteomic experiments suggested K73 as the modification site shared by ubiquitylation and acetylation, and its role was confirmed using K73R mutant CTH. Additional results showed honokiol binding to HDAC6, producing an additive effect with tubastatin A. Therefore, it was suggested that honokiol attachment may impede the substrate’s accessibility to the deacetylase. Additionally, in lung cancer cell lines, CTH ubiquitylation by Rad18 and the REV1 scaffolding role were indicated by the effects of ligase silencing, REV1 overexpression, and MG132 addition [156]. In fact, REV1 levels were also upregulated by proteasomal inhibition and its deubiquitylation by USP9X [156].

Other pieces of evidence regarding ubiquitylation of sulfur amino acid metabolism enzymes have been obtained from *C. elegans*, where the interaction of AHCY1 with the E3 ubiquitin ligase CHN-1 was identified [160], although no reports on equivalent mammalian proteins have been published. Interestingly, research comparing the BHMTs of 62 species highlighted the conservation of ubiquitylation sites, except for K98^M^ which becomes E98 in the human protein [63]. Moreover, as this is also a succinylation site, its substitution suggests a change in the regulatory mechanisms controlling human BHMT and/or its interaction network, and this deserves further examination.

### 5.2. Regulation by Sumoylation

HTP data about sumoylation of enzymes of sulfur amino acid metabolism are restricted to the identification of a single site in AHCY, obtained using cell lines (Table 5), although other type of studies have provided information concerning MATα1, MATα2, MATβ, CBS, and CTH. Liver samples of both human alcohol-induced steatosis and ethanol-fed mice exhibited enhanced MATα1 sumoylation compared with control samples, which was further confirmed by anti-SUMO 2/3 immunoprecipitation and proximity ligation assays [161]. In primary hepatocytes, *Sumo2* silencing preserved MATα1–TOM20 interaction and, in turn, MATα1 mitochondrial localization [161]. K48 was identified as the sumoylation site, and the corresponding K48R mutant was shown to decrease MATα1–PIN1 interaction while increasing MATα1–TOM20 binding in vehicle-treated primary mouse hepatocytes. In contrast, ethanol induced not only MATα1 sumoylation but also serine phosphorylation favoring MATα1–PIN1 interaction and, subsequently, preventing MATα1 mitochondrial translocation [161]. As previously mentioned, S114 phosphorylation by ethanol-activated CK2 increased MATα1–PIN1 interaction and MATα1 cytoplasmic retention [127]. These effects were reduced in the K48R mutant, which exhibited higher stability than wild-type MATα1 in cycloheximide chase experiments and upon treatment with MG132 and bafilomycin [161]. Moreover, these assays also showed ethanol-induced MATα1 degradation via both the proteasomal and autophagy pathways and decreased ubiquitylation of the K48R mutant, thus suggesting that sumoylation was required for ubiquitylation and proteasomal degradation [161]. The authors also used gene editing to introduce the K48R mutation in mouse primary hepatocytes and in the NIAAA model, which led to protection from ethanol-induced consequences such as mitochondrial dysfunction and fat accumulation while reducing MATα1–PIN1 interaction and increasing MATα1 phosphorylation levels [161].

Diverse studies have shown upregulation of *UBC9*, *BCL2*, and *MAT2A* expression in cancer cells. The three proteins converge in the regulation of apoptosis, which is inhibited by the E2 ligase Ubc9 and promoted by AdoMet-induced decreases in *UBC9* and *MAT2A* expression [162]. MATα2 binds at three sites on the Bcl-2 promoter, activating its transcription; this effect is independent of AdoMet synthesis as it is also produced by inactive MATα2 mutants [162]. Additionally, coimmunoprecipitation showed MATα2–Bcl-2–Ubc9 interaction and a decrease in MATα2–Bcl-2 binding upon *UBC9* silencing. MATα2–Bcl-2 interaction enhanced Bcl-2 stability, an effect dependent on the stability of MATα2 itself, linked to its Ubc9-dependent sumoylation on K340, K372, and K394. SUMO-1 modifies cytoplasmic and nuclear MATα2 in cells, and the level of sumoylation is decreased by silencing of *UBC9* or *SUMO1* [162]. Interestingly, nuclear MATα2 modification levels seem higher than those in the cytoplasm. Moreover, protection from drug-induced apoptosis in cancer cells is attained only with wild-type MATα2 overexpression and not with mutations on the sumoylation sites. Based on these results, Tomasi et al. proposed that Ubc9-dependent defense from apoptosis may occur through MATα2 stabilization upon its sumoylation and its subsequent upregulation of Bcl-2 transcription [162]. Decreased sumoylation in Ubc9-depleted HeLa cells was also related to an increase inMATβ in the nuclear matrix subcellular fraction, in which label-free proteomics revealed high sumoylated protein levels [163]. However, no MATβ sumoylation sites were reported.

**Table 5:** Other modification sites in enzymes of the mammalian methionine cycle and reverse transsulfuration, identified using high-throughput approaches.

PTM [Ref] ^4^	Gene Name	Modification Site [Ref] ^5^
Sumoylation ^1^ (cell lines) [57]	*AHCY*	**K**322
N-glycosylation (mouse^L^) [101]	*MAT1A*	**N**106
	*AHCY*	**N**126, **N**181
	*BHMT*	**N**69
Monomethylation (cell lines)	*MAT1A*	**R**264 [64,65]
	*MAT2A*	**R**192 [65]
	*MAT2B*	**R**29 [65], **R**30 [65]
	*AHCY*	**K**8 [66], **R**19 [65], **R**34 [65], **R**123 ^2^ [65], **R**205 [65], **R**299 ^2^ [65], **R**301 ^2^ [65], **R**403 ^2^ [65]
	*MTR*	**K**817 [66], **R**1132 [65]
	*CBS*	**R**18 [65], **R**164 [65], **R**190 [65], **R**336 [65], **R**389 [65]
	*CTH*	**R**62 [65], **R**119 [65], **R**122 [65]
Succinylation ^3^ (mouse^L^)	*MAT1A*	**K**48 [164], **K**54 [164], **K**98 [59], **K**392 [59]
	*AHCY*	**K**8 [63], **K**43 [59], **K**204 (cells, mouse^L^) [59], **K**322 [59], **K**389 [59], **K**401 [164], **K**405 (cells, mouse^L^) [59,164], **K**408 [164]
	*GNMT*	**K**46 [63], **K**191 [63], **K**196 [59,63], **K**201 [59,63], **K**238 [59,63]
	*MTR*	**K**327 [59]
	*BHMT*	**K**7 [164], **K**8 [164], **K**10 [164], **K**11 [164], **K**35 [164], **K**40 [63], **K**93 [59,63], **K**98 [63,164], **K**139 [164], **K**150 [59], **K**207 [63,164], **K**232 [59,63], **K**241 [63], **K**283 [59,63], **K**340 [63], **K**349 [59,63], **K**377 [63], **K**386 [59,63]
	*BHMT2*	**K**274 [59,63]
	*CBS*	**K**485 (cells) [59], **K**174 [59]
	*CTH*	**K**138 [63], **K**270 [59], **K**325 [63]
Malonylation (mouse^L^) [107]	*MAT1A*	**K**89, **K**235
	*AHCY*	**K**4, **K**20, **K**389, **K**408
	*GNMT*	**K**148, **K**159, **K**191, **K**196, **K**201, **K**275
	*BHMT*	**K**11, **K**82, **K**93, **K**98, **K**150, **K**207, **K**229, **K**232, **K**241, **K**283, **K**349, **K**377, **K**386
	*CTH*	**K**72, **K**140, **K**329
Lactylation (human^L^) [106]	*MAT1A*	**K**53, **K**88, **K**91, **K**223, **K**234, **K**265, **K**285, **K**351, **K**367, **K**373
	*AHCY*	**K**20, **K**186, **K**188, **K**226, **K**322, **K**388, **K**389, **K**401, **K**405
	*GAMT*	**K**109
	*GNMT*	**K**193, **K**198, **K**203
	*BHMT*	**K**40, **K**93, **K**139, **K**150, **K**207, **K**226, **K**232, **K**241, **K**283, **K**340, **K**349, **K**369, **K**386, **K**395, **K**400
	*BHMT2*	**K**104, **K**120, **K**198
	*CBS*	**K**25, **K**30, **K**177, **K**281
	*CTH*	**K**73, **K**139, **K**141, **K**260, **K**304
Hydroxybutyrylation (mouse^L^) [105]	*AHCY*	**K**20, **K**43, **K**188, **K**204, **K**389, **K**405
	*GNMT*	**K**159, **K**191, **K**196, **K**201, **K**275
	*BHMT*	**K**40, **K**93, **K**98, **K**207, **K**232, **K**241, **K**349, **K**377, **K**386
	*CTH*	**K**140
Methylglyoxal (Cell lines) [67]	*MAT2A*	**C**56, **C**104 ^6^, **C**214
	*MAT2B*	**C**58 ^6^, **C**297 ^6^
	*AHCY*	**C**195 ^6^, **C**228, **C**421 ^6^
	*GAMT*	**C**16, **C**91 ^6^, **C**169 ^6^, **C**220 ^6^
	*PEMT*	**C**70 ^6^
	*CBS*	**C**15 ^6^, **C**52, **C**103, **C**109, **C**165, **C**370, **C**431
	*CTH*	**C**70, **C**109, **C**137, **C**229
Formaldehyde (mouse^L^) [108]	*MAT1A*	**C**105, **C**121, **C**150, **C**376 ^7^
	*AHCY*	**C**79, **C**113, **C**195, **C**278
	*GAMT*	**C**92, **C**169, **C**185, **C**220, **C**236
	*GNMT*	**C**147, **C**186, **C**247
	*BHMT*	**C**104, **C**131, **C**217, **C**256, **C**299, **C**300
	*BHMT2*	**C**290, **C**391
	*CBS*	**C**427

^1^ WaLP proteolysis and validation by desumoylation with SENP1 and SENP2 and deubiquitylation with Usp2cc. ^2^ Residue number in the AHCY isoform 2 that lacks the 1–28 sequence of canonical AHCY. ^3^ Residues modified in cells or in both mouse liver and cells are indicated in parentheses following the amino acid number. ^4^ Reference number for single studies. ^5^ Reference numbers when more than one study reported the modification. ^6^ Found in more than one cell line or lysate. ^7^ Found only in recombinant MATα1.

Several proteins involved in the sumoylation machinery were identified as CBS interaction targets during the screening of a human brain library [23]. Precisely, these included the Ubc9-conjugating enzyme, the ligases PIAS1, PIAS3, and hPc2, and the RanGTPase-binding protein RanBPM, all of which bind to the CBS C-terminal regulatory domain [23]. Moreover, SUMO-1 modification of CBS has been observed in vitro and in vivo, where the modified protein changed its subcellular localization towards the nuclear scaffold [23]. Using a set of CBS mutants, K211R was shown to preclude incorporation of SUMO. It is noteworthy that this residue is included in a putative sumoylation motif that is exposed according to the protein structure. CBS nuclear localization has been confirmed using several techniques and this translocation relates to its sumoylation, although subcellular fractionation and immunoblotting demonstrated the nuclear occurrence of both sumoylated and unmodified forms [23]. Additional in vitro studies with the SUMOlink kit (containing Uba2, Ubc9, and SUMO-1) showed enhanced CBS sumoylation in the presence of the SUMO E3 ligase hPc2 (a component of the repressive PRC1 complex) [165]. The absence of hPc2 resulted in decreased modification when the enzyme substrates Hcy, serine, or cysteine or the product cystathionine were included in the assay [165]. Interestingly, AdoMet exerted no further effect. In contrast, when hPc2 and the substrates and product were combined, the inhibitory effect of cystathionine on CBS sumoylation was precluded. Moreover, in vitro sumoylation inhibited CBS activity, and this effect was enhanced in the presence of hPc2 [165]. Similar in vitro assays using recombinant CTH demonstrated that its putative sumoylation was not affected by the cystathionine substrate [165].

Again, as for other already mentioned PTMs, sumoylation seems to be especially involved in the subcellular localization of enzymes associated with sulfur amino acid metabolism.

## 6. Posttranslational Modification by Products of Several Metabolic Pathways

Metabolites of glycolysis, the TCA cycle, and fatty acid synthesis can be used in their CoA-activated forms for the modification of proteins. Nonetheless, the list of routes providing substrates for PTMs is broader, including, e.g., the polyamine synthesis pathway. Several of these PTMs have been identified in enzymes associated with sulfur amino acid metabolism, although their functional impact has not been studied in depth.

### 6.1. Regulation by Acetylation

Acetylation of proteins has been examined in several HTP studies using cell lines (Table 1) or mammalian tissues (Table 2), where many modification sites have been identified in enzymes of sulfur amino acid metabolism (Table 6). However, systematic exploration of N-α-acetylation that frequently occurs at the initial position of eukaryotic proteins is not so common. Among the main enzymes of the methionine cycle and reverse transsulfuration, S2-acetylation of GAMT was identified in a large study using human A2780 ovarian cancer cells [62], although no functional information was provided. Other authors described differences in N-terminal acetylation between purified rat liver and recombinant GNMTs, identified on V2 of the liver protein [139]. More recently, N-terminal acetylation of MATα1, MATα2, AHCY, GAMT, MTR, and PDRG1 has also been reported [51].

Initial reports concerning AHCY described its acetylation on K401 and K408, although their functional relevance was again not elucidated [61]. Later, a semisynthetic approach was used to produce single and diacetylated C-terminal AHCY peptides (396–432) that were fused to the rest of the protein (E396C mutant) for intein-tagged expression [166]. The resulting wild-type and acetylated forms preserved the homotetrameric association state, but k_cat_ values decreased in the modified forms and K_m_^AdoHcy^ also increased in the diacetylated form [166]. The effects on catalysis were confirmed by site-directed mutagenesis, and the crystal structures of K401ac and K408ac AHCY forms were obtained in the presence of NAD^+^. These structures showed the low electron density of the modified side chains and alteration of the hydrogen bonding pattern [166].

Acetylation of AdoMet synthesis enzymes has been detected in *E. coli* MAT, as well as in mammalian MATα2. In the purified bacterial enzyme, up to 14 lysine residues were modified, some resulting from in vitro autoacetylation [167]. The same work described decreased tripolyphosphatase activity of acetylated *E. coli* MAT and several K/R or K/Q mutants in vitro, while deacetylation by the NAD-dependent deacylase CobB restored enzyme activity. Other work carried out with cell lines identified acetylation of MATα2 and its increase upon trichostatin A inhibition of histone deacetylases [154]. K81 was the modified residue identified by mass spectrometry and its role was confirmed using K81R and K81Q mutants, *MAT2A* knockdown and acetylated K81 peptides. As already mentioned, K81 modification was folate-dependent, deprivation resulting in enhanced MATα2 acetylation and decreased protein levels in HEK293T and Huh7 cells. These effects were preventable through the combination of MG132 with either trichostatin A or folate deprivation, while the proteasomal inhibitor alone led to MATα2 accumulation in a variety of cell lines [154]. In fact, that half-life of MATα2 decreased in the presence of trichostatin A and folate deprivation, while these effects were prevented in the K81 mutants. Altogether, these results suggest induction of proteasomal degradation by acetylation. Immunoprecipitation from HEK293T coexpressing MATα2 and several histone acetyltransferases or deacetylases revealed MATα2–P300 and MATα2–HDAC3 interactions, the former being enhanced under folate deprivation [154]. As expected, interactions of MATα2 with P300 and HDAC3 increased and decreased levels of K81 acetylation, respectively. These results were reinforced by analogous experiments carried out with MATα2 K81R and K81Q mutants expressed in P300- or HADC3-silenced cells. Furthermore, stable *MAT2A* knockdown in HepG2 cells led to arrest of growth, while reexpression of wild-type MATα2 or its K81 mutants under folate deprivation resulted in faster proliferation [154]. Cells carrying the MATα2 mutants had no alteration of their AdoMet–AdoHcy ratio or global methylation levels [154]. Xenografts of HepG2 cells from mice fed a folate-free diet showed the same behavior as cells overexpressing MATα2 mutants. In contrast, only 50% of the human HCC samples examined showed increased MATα2 levels versus adjacent normal tissue, while a lower percentage presented increased K81 acetylation [154]. Therefore, inverse correlation between K81 acetylation and MATα2 protein levels were indicated and, as mentioned in previous sections, crosstalk with ubiquitylation was observed to take place at this residue. The crystal structure shows K81 located in the central domain through which dimers interact to form the tetramer; hence, an additional consequence of its modification may be the dissociation of the oligomer to facilitate degradation. Remarkably, K81 is substituted by R81 in MATα1, thus suggesting distinct regulatory effects of this position in both catalytic subunits.

A recent study also showed decreased MATα2 stability by *MAT2B* knockout, a context in which its global acetylation and MATα2-P300 binding were increased [168]. Deletions of the MATβ cofactor motif and variations in NADP^+^ levels (NADK overexpression or enhanced consumption through the pentose phosphate pathway) demonstrated that this stabilization effect was NADP^+^-dependent. Additionally, results from cultures under high glucose with inhibitors of glycolysis or pyruvate, which also changed NADP^+^ levels, further indicated this dependency on NADP^+^. Interestingly, inhibition of glycolysis due to MATα1 depletion was reported in NSCLC cells lines [169], and this regulatory mechanism seemed to involve its competition with CCND1 for E3 ligase SKP2 binding.

### 6.2. Regulation by Glycosylation

Different types of glycosylation have been identified in PEMT and AHCY, whereas no information appears to be available for the other enzymes of interest relating to mammalian sulfur amino acid metabolism (Table 5). Expression in HEK293 cells of the long and short forms of PEMT with different N-terminals revealed higher activity of the latter and differences in their substrate specificities [170]. Using a set of deglycosylases, changes in the electrophoretic mobility of the long PEMT were demonstrated, suggesting its modification with high-mannose oligomers according to the enzyme’s susceptibility. The modification site predicted was N13, located in the N-terminal segment that is lacking in the short PEMT. This segment lies on the luminal ER side in the available topological PEMT models [170]. Based on these results, Morita et al. proposed that N-glycosylation of the long PEMT form regulates both specificity and enzyme activity.

O-linked β-N-acetylglucosamine (O-GlcNAcylation) on mouse AHCY was found to be critical for embryonic stem cell (ESC) pluripotency, in a series of experiments that analyzed its modification levels during LIF-minus or retinoic acid-induced differentiation [171]. During mESC differentiation, AHCY O-GlcNAcylation was decreased, as were *AHCY* and *MAT2A* expression, AHCY protein content, and metabolic flux through the methionine cycle. Further analysis in E14.1 cells after *Ahcy* silencing showed a correlation with decreased pluripotent markers, ribosomal protein expression, and H3K4me3 epigenetic methylation at *Oct4* and *Nanog*, together with an increase in the percentage of apoptotic cells [171]. Modification on T136, T141, T185, and S187 was identified using immunoprecipitates from cells coexpressing tagged AHCY and OGT and LC-MS/MS; T136 was recognized as the main O-GlcNAcylation site in assays with alanine mutants [171]. Additionally, immunoprecipitated AHCY appeared preferentially as tetramers which, upon T136 modification, increased their AdoHcy-hydrolyzing activity by enhancing their affinity for this substrate [171]. The authors also showed that AHCY protein and O-GlcNAcylation levels were even lower in MEFs than in ESCs; these parameters were induced by reprograming with Yamanaka factors [171].

### 6.3. Regulation by Amidation and Crosslinking

This type of modification has been identified only in BHMT, where it was found among substrates of liver transglutaminase using 5-(biotinamido) pentylamine as a probe, with avidin purification and N-terminal sequencing [172,173]. Transglutaminases catalyze amine incorporation and crosslinking as well as glutamine deamidation in protein targets. In vitro assays using tissue-type transglutaminase confirmed incorporation of histamine, putrescine, or spermidine on BHMT, while detection of ammonia release in the absence of primary amines suggested glutamine crosslinking (intra- and inter-subunit) or deamidation [172]. The presence of crosslinked BHMT dimers and tetramers was later confirmed using immunoblotting and gel filtration chromatography [172]. Although both crosslinking and deamidation decreased BHMT activity, the effects of crosslinking seemed stronger. After biotin labeling, avidin capture, and MALDI-TOF analysis, several degrees of modification were detected on a porcine BHMT peptide (382–396) [172]. This C-terminal peptide includes four glutamines and is situated at the center of a long α-helix, with substantial surface contact with the subunit located immediately below or above in the BHMT tetramer holding the structure together [174]. The four glutamines in this α-helix face the surface with their lateral chains exposed, thus making crosslinking with nearby subunits of another tetramer feasible. Interestingly, BHMT2 lacks this part of the sequence.

### 6.4. Regulation by Malonylation

**Figure 5:**
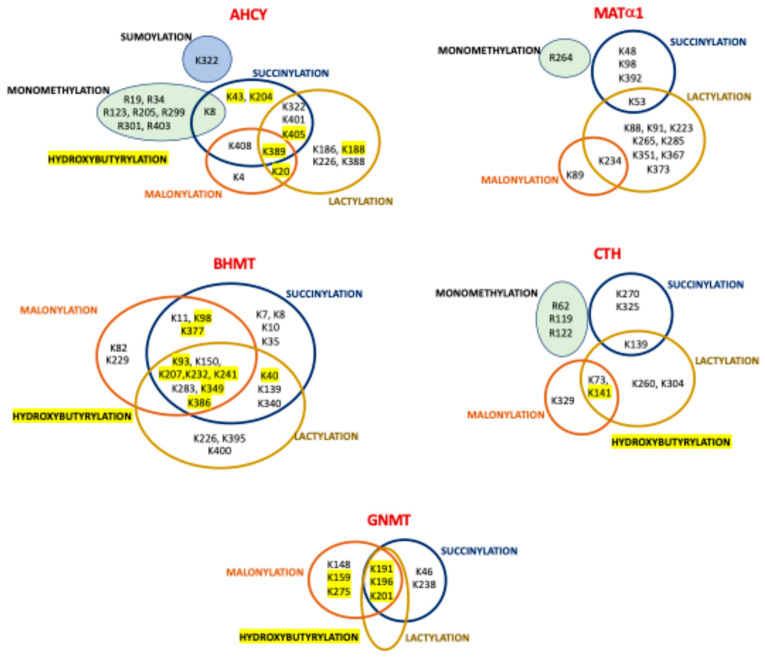
Overlap between other modifications targeting basic residues. Limited overlap between basic residues that are targets of monomethylation (green background circle), succinylation (blue circle), lactylation (gold circle), malonylation (orange circle), and hydroxybutyrylation (highlighted in yellow) has been identified in high-throughput studies, as shown in the figure. In fact, only a few residues are shared among these PTMs in AHCY, GNMT, MATα1, BHMT, and CTH, while no such overlap has been identified in other enzymes of mammalian sulfur amino acid metabolism. Residues identified as targets of each PTM are indicated in the corresponding circles. The single sumoylation site found in AHCY is also depicted. Abbreviations: AHCY, S-adenosylhomocysteine hydrolase; BHMT, betaine homocysteine S-methyltransferase; CTH, cystathionine γ-lyase; GNMT, glycine N-methyltransferase; MATα1, methionine adenosyltransferase α1 catalytic subunit.

The impact of SIRT5 on lysine malonylation was evaluated using anti-malonyl lysine enrichment in the livers of wild-type and *Sirt5^−/−^* mice [107]. HTP study identified several malonylation sites in enzymes of interest (Table 5), some of which were sample-specific (Figure 5). BHMT malonylation on K82, K93, K377, and K386 was found only in knockout livers, whereas BHMT K283, AHCY K408, and CTH K329 were modified only in the wild-type samples [107]. Quantification of the KO–wt malonylation ratio indicated substantial increases for three BHMT sites (K386, K377, and K93) and one GNMT (K196), whereas a decrease was observed with AHCY K20 modification [107]. No data on the functional relevance of the malonylation of these enzymes was provided although, interestingly, in *Sirt5^−/−^* livers, the highest modification levels were identified in AldoB, a BHMT interaction target [175]. Nevertheless, the effect of AldoB–BHMT interaction on the respective enzyme activities and the impact of malonylation on this interaction remains unexplored.

### 6.5. Regulation by Lactylation

The Warburg effect exhibited by many cancer cells favors their accumulation of lactate, a metabolite that in the form of lactyl-CoA serves as substrate for protein lysine lactylation, a modification initially described in histones. However, new studies using HBV-related HCC and adjacent liver tissue have uncovered a wider impact of lactylation, especially in metabolic enzymes [106]. These include AHCY, BHMT, BHMT2, GNMT, MATα1, GAMT, CBS, and CTH, in which a variable number of lactylation sites have been identified via HTP study (Table 5). Interestingly, several of the lactylation sites identified in AHCY, BHMT, GNMT MATα1, and CTH are also targets of succinylation, malonylation, and hydroxybutyrylation. Of note, the highest overlap was detected for BHMT, in which six lysines can be targets for the four PTMs (Figure 5). Yang et al. also described a general increase in lactylated proteins and in the number of modified sites per protein in tumor samples versus adjacent tissues, an overlap of lactylation and acetylation sites that reached ~56% for metabolic enzymes, and an enrichment of lactylated proteins in the cytosol [106]. Furthermore, correlation between worse prognosis in HCC and increased protein lactylation levels was also described. Global lactylation was decreased by inhibition of P300 and enhanced by *HDAC3* knockout, representing the writer and eraser of the modification, respectively [106]. Although the functional effects of lactylation on enzymes of the methionine cycle and reverse transsulfuration remain unknown, the related enzyme adenosine kinase 2 (ADK2) was inhibited by its lactylation on K28, leading to increased proliferation and metastasis of HCC cells [106]. As ADK is mainly responsible for adenosine elimination in adult hepatocytes, its inhibition would make clearance of AdoHcy mostly dependent on Hcy catabolism and export. Therefore, it is tempting to suggest that lactylation of other enzymes involved in the hepatic methionine cycle may also reduce their activities in order to avoid AdoHcy accumulation, although there is currently no evidence to support this hypothesis.

### 6.6. Regulation by Succinylation

Succinylation is related to carbon stress and occurs mainly in a non-catalyzed manner, although some succinyltransferases have been recently identified. Conversely, succinyl and malonyl groups in proteins are known to be removed by SIRT5, a mitochondrial and cytoplasmic enzyme [63]. Hence, HTP studies on succinylation have analyzed livers [63,164] and SILAC-labeled MEFs [63] of wild-type and *Sirt5^−/−^* mice, using different proteolysis protocols (Table 1 and Table 2). Nevertheless, both of these studies identified almost the same modification sites (Table 5), and Zhang et al. also quantified the KO–wt stoichiometric ratio of succinylated sites [164]. This ratio was elevated in a few cases, suggesting their regulation by SIRT5, e.g., K48 in MATα1 with a KO–wt ratio > 5 [164]. Moreover, in addition to MATα1, succinylation sites were also identified in AHCY, GNMT, MTR, BHMT, BHMT2, CBS, and CTH (Table 5), showing different degrees of overlap with other modifications targeting lysines (Figure 5). The authors paid special attention to the coincidence of succinylation and acetylation sites (Table 5 and Table 6), which was more restricted than in the study by Weinert et al. [59,63,164]. Interestingly, although BHMT and GNMT presented the highest numbers of modification sites, no functional information about the impact of succinylation was provided. Nevertheless, we can guess that the succinyl group may alter the microenvironment surrounding the modified lysine, due to its size and/or the change of charge introduced in the lateral chain. Analysis of the position of succinylated residues in the BHMT crystal structure suggests that, for example, modifications on K386, K377, K349, and K283 may affect the tetramer’s stability due to their location on the C-terminal α-helix or just below this monomer–monomer fastening element. Additional implications of succinylation may be related to its regulation of the urea cycle [164] and, in turn, of polyamine synthesis, which also depends on AdoMet synthesis by MATα1 homo-oligomers that are also susceptible to this modification.

### 6.7. Regulation by Hydroxybutyrylation

The main ketogenic body produced in several dietary regimes and diseases is β-hydroxybutyrate, which in the form of β-hydroxybutyryl-CoA serves as substrate for lysine modification. This PTM was analyzed in a HTP study that used a variety of mouse models with increased β-hydroxybutyrate levels (e.g., starvation, ketogenic diet, streptozotocin) and cell lines treated with the sodium form of this metabolite [105]. Examination of mouse tissues revealed enhanced hydroxybutyrylation levels only in liver and kidney proteins [105]. Additionally, starved mouse livers were used for the identification of modification sites (Table 2), and the overlap between acetylation and hydrobutyrylation in their mitochondrial fractions was analyzed. The increased acetyl-CoA production that occurs during ketogenesis subsequently results in β-hydroxybutyrate synthesis. Although several hydroxybutyrylated residues on AHCY, GNMT, BHMT, and CTH were detected (Table 5), only AHCY modification was examined further. Starved mice and those on a ketogenic diet presented increased AHCY hydroxybutyrylation but no changes in acetylation levels [105]. Expression of tagged AHCY in MEFs treated with vehicle or sodium β-hydroxybutyrate allowed detection of the modification, whose levels were inversely correlated with enzyme activity. Moreover, starved mouse livers also exhibited decreased AdoHcy clearance and enhanced levels of this metabolite [105]. The presence of four AHCY hydroxybutyrylated sites (K188, K204, K389, and K405) close to the interface between the NAD^+^ cofactor and substrate binding sites in the NAD^+^-bound AHCY tetramer structure prompted their mutation and analysis in HEK293T cells. The K188R mutant already exhibited reduced enzyme activity without treatment, whereas the K389R and K405R substitutions impaired the inhibition induced by sodium β-hydroxybutyrate [105]. Therefore, it was deduced that hydroxybutyrylation interferes with key interactions of these residues to stabilize the NAD^+^ binding site. Moreover, the inhibitory effect of β-hydroxybutyryl-CoA on recombinant AHCY was concentration-dependent in the absence of any acylating enzyme [105]. As already mentioned, several hydroxybutyrylated lysines identified in AHCY, GNMT, BHMT, and CTH are the targets of additional modifications (Figure 5), their functional impact remaining mostly unexplored.

### 6.8. Regulation by ADP-Ribosylation

Knowledge of the mechanism regulating tumor cells’ dependence on methionine for growth, known as the Hoffman effect, is of clinical importance. Hence, animal and cell models subjected to methionine restriction have been used for the elucidation of this phenomenon, especially in the context of liver carcinogenesis. Such models have shown extensive impact on gene expression and the metabolome, and pathway analysis has revealed alteration of genes involved in nutrient-sensing routes (i.e., mTOR) and their downstream oncogenic transcription factors [176]. Among them, c-Myc binds to E-box elements, increasing promoter activities such as that of *MAT1A* when associated with MafG and c-Maf, a complex that can also include MATα1 in cases of cholestatic livers and cholangiocarcinoma [177]. Methionine restriction downregulated c-Myc and reduced phosphorylation of p70 S6K and TRIM32, while upregulating SIRT4 and delaying tumor growth [176]. Although no changes in MATα2 were found, SIRT4 overexpression in cells decreased AdoMet, AdoHcy, Hcy, and histone methylation levels [176]. Previous works reported potential SIRT4–MATα2 interaction as well as the role of SIRT4 as a mono-ADP-ribosylation (MARylation) enzyme. Hence, the effects of changes in *SIRT4* expression on MATα2 MARylation were analyzed, and the results showed enhanced MATα2 MARylation with SIRT4 overexpression and decreased modification upon *SIRT4* silencing, c-Myc induction, or SIRT4 inhibition with nicotinamide and sirtinol [176]. Altogether, c-Myc overexpression resulted in enhanced MATα2 activity and AdoMet production, which were linked to TRIM32-dependent ubiquitylation of SIRT4 and its proteasomal degradation. Interestingly, HCC patient samples also exhibited increased c-Myc, with low SIRT4 and MATα2 MARylation. Furthermore, alanine mutational screening of potential MATα2 MARylation sites revealed E111 to be the critical modification site [176]. This residue is situated next to Q113 at the MATα2 gating loop [178], for which role in methionine positioning for catalysis has been proposed.

### 6.9. Regulation by Methylglyoxal

**Figure 6:**
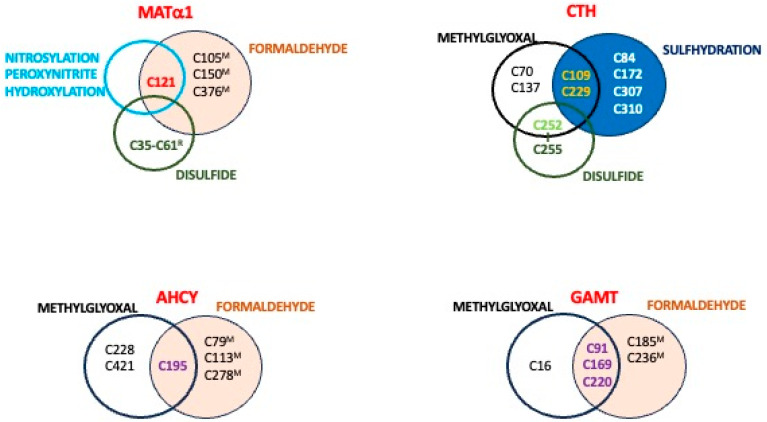
Overlap between cysteine residues targeted by several redox modifications. Limited overlap between residues targeted by redox modification is observed in enzymes of sulfur metabolism, including MATα1, AHCY, GAMT, and CTH, as shown in the figure. Residues identified for each of the modifications depicted are included in the colored circles as follows: methylglyoxal targets (black); formaldehyde (salmon background); disulfide bonds (dark green); nitrosylation, hydroxylation and peroxidation targets (light blue); and sulfhydration (blue background). Overlapping residues are indicated with colored bold font as follows: nitrosylation and formadehyde overlap (red); formaldehyde and methylglyoxal targets (purple); methylglyoxal and sulfhydration overlaps (yellow); disulfide bond (dark green) and glutathionylation targets (light green).

Glucose metabolism is the main producer of methylglyoxal, which can reach intracellular concentrations up to 300 μM. Its binding to proteins or glutathione is mostly reversible in the form of hemithioacetals. Lysates of three cancer cell lines were used in the search for modified cysteines, including HeLa cells incubated with methylglyoxal for SILAC-based competitive iodoacetamine–alkyne profiling [67]. Among them, HEK293 lysates showed a wider array of modified enzymes of interest, including MATα2, MATβ, AHCY, GAMT, PEMT, CBS, and CTH. Importantly, modification of residues C195 and C421 of AHCY and C91 of GAMT was identified under all conditions [67], although the SILAC ratio was <2.5 fold, the limit chosen for cysteine susceptibility. Conversely, higher ratios were displayed by C58 of MATβ, C229 of CTH, and C220 and C169 of GAMT only in certain samples [67]. The functional effects on these enzymes were not explored and, based on the structural information, only some deductions can be made. For example, differences in the exposure of C195 and C412 lateral chains were observed in the AHCY tetramer, the latter being exposed at the surface, easily facilitating its modification. Importantly, to date, limited overlap has been identified between cysteine residues targeted by methylglyoxal and other PTMs in sulfur amino acid metabolism enzymes, as summarized in Figure 6.

### 6.10. Regulation by Formylation

Formaldehyde is an electrophilic compound generated both endogenously and upon exposure to exogenous compounds. The formate produced after its oxidation can be incorporated into the folate cycle, providing carbon units to be used in related pathways such as the methionine cycle. Proteins containing cysteine residues sensitive to formaldehyde were identified in a proteomic study carried out with mouse liver lysates subjected to this agent, including MATα1, GNMT, AHCY, BHMT, GAMT, and CBS, along with enzymes involved in glutathione synthesis or in serine/glycine metabolism [108]. Moreover, comparison of data from mouse liver lysates treated with vehicle or formaldehyde concentrations close to those measured in disease (500 μM) enabled HTP identification of the modified residues (Table 5). Three cysteines in MATα1 (C105^M^, C150^M^, and C121^M^), two in BHMT (C104^M^ and C131^M^) and one each in AHCY (C278^M^), GNMT (C186^M^), GAMT (C91^M^), and CBS (C427^M^) were detected with treated–untreated ratios > 3 [108]. Remarkably, some of these residues are also targets of other PTMs (Figure 6), thus putatively suggesting crosstalk.

Formaldehyde modification of MATα1 led to its inactivation and a decrease in AdoMet production that was prevented in the human C120S mutant [108], in an effect that was expected from the location of this cysteine at the loop regulating the access of substrates to the active site. This type of regulation seems to be specific to MATα1 isoenzymes and context-dependent, as the equivalent cysteine is lacking in MATα2 and its introduction was not enough to obtain formaldehyde-dependent inhibition [108]. Moreover, physiological levels of formaldehyde exerted stronger inhibition of MATα1 than H_2_O_2_ or NO, both of which also target C120 of MATα1. In vivo, *Adh5^−/−^* mice with chronic formaldehyde elevation due to its impaired removal also showed decreased AdoMet production but increased MATα1 protein levels and hypomethylation of the *Mat1a* promoter and specific histone positions [108]. Knockdown of transcription factors regulating the *MAT1A* promoter allowed association of formaldehyde’s effects on expression with the activity of HNF4α, C/EBPα, and C/EBPβ [108]. Altogether, formaldehyde seems to exert global regulation of methionine and cysteine metabolism that, at least for MATα1, takes place at many levels. Thus, more studies are needed to fully understand the regulation of these pathways by formylation on other enzymes.

## 7. Redox Regulation and Associated Posttranslational Modifications

Together with phosphorylation, redox regulation of sulfur amino acid metabolism was one of the first mechanisms explored in several specific studies in which modifications at single sites as well as the existence of disulfide bonds were uncovered. This interest arose from data showing impaired function of the methionine cycle, especially of AdoMet synthesis, under pathological conditions correlating with redox stress such as cirrhosis [179]. The use of several animal and cell models in which free radical production was induced also showed oxidative inactivation of AdoMet synthesis (e.g., CCl_4_, paracetamol, and LPS intoxication), which correlated with alterations in GSH levels in some cases (e.g., CCl_4_, paracetamol, and buthionine sulfoximine (BSO) treatments) [34,179,180,181,182,183]. Moreover, in vitro incubation of purified recombinant or liver MAT I/III with oxidants (e.g., H_2_O_2)_, nitric oxide (NO) donors, and N-ethylmaleimide (NEM) also resulted in loss of enzyme activity [182,183,184,185,186]. In certain cases, these conditions also induced dissociation of oligomers into inactive MAT III dimers (e.g., cirrhosis, galactosamine and BSO treatments) and monomers (GSSG incubation) [34,179,180].

Further exploration of the effects of these treatments uncovered the regulatory role of the GSH–GSSG ratios in AdoMet synthesis using purified rat liver MAT I/III [187] and the ability of thiolreductases to maintain these effective ratios within physiological levels [188]. As discussed below, additional data from these in vitro experiments suggested thiol–disulfide redox regulation of MAT I/III. On the other hand, the outcomes derived from NO and H_2_O_2_ treatments in vitro and in cells were reversible and, in turn, MAT I/III activity was recovered [182,183]. Interestingly, effects derived from H_2_O_2_ treatment of MAT I/III were prevented by the Fe^2+^ chelator desferoxamine, but each isoenzyme required a distinct GSH concentration for reactivation; those needed by MAT I were well above the GSH physiological range. Moreover, C121 was identified as the target of hydroxyl radicals [183] and S-nitrosylation [186], but the latter modification was found to be context-dependent and its introduction relied on the nearby residues D355, R357, and R363 [186]. According to the crystal structure of MAT 1, C121^R^ locates at the flexible loop regulating the access of substrates to the active site, and hence, its modification could block the loop in a closed conformation that precludes AdoMet synthesis. As already mentioned, this residue is not conserved in MATα2, in turn allowing differential regulation among MAT isoenzymes (Figure 6). Enhancement of endogenous H_2_O_2_ levels in HUVEC cells or its exogenous addition has also been reported to increase CTH activity and, subsequently, H_2_S levels [189]. These effects were prevented by H_2_O_2_ scavengers. Unfortunately, no identification of the putative CTH residues involved was carried out.

Paracetamol overdose models are known to cause hepatotoxicity in a process that involves GSH depletion during drug catabolism and generation of the reactive NAPQI metabolite. Protein modification can result directly from NAPQI binding or indirectly by metabolites produced through the associated oxidative stress. Among the latter, modification of BHMT by 4-hydroxynonenal (4-HNE) was reported in APAP-intoxicated mouse livers by means of LC-MS/MS identification of proteins in anti-4-HNE positive spots [190]. However, the specific modification site was not reported, and neither was the functional effect of this incorporation. It is noteworthy that nuclear accumulation of MATα1 was found in APAP-induced acute hepatic injury, and the associated GSH depletion also induced changes in the nucleocytoplasmic distribution of several enzymes assiociated with methionine metabolism [34]. However, the putative PTMs involved in these localization changes were not analyzed.

### 7.1. Disulfide Bonds

N-ethylmaleimide (NEM) labeling of purified rat liver MAT I/III revealed a difference in the number of labeled cysteines between denatured proteins in the absence or presence of DTT [184,191], suggesting the presence of a disulfide bond. Further experiments combining NEM labeling, peptide mapping, amino acid analysis, and N-terminal sequencing assigned C35^R^ and C61^R^ as the residues involved in the intrasubunit disulfide [191]. Moreover, the orientation of both side chains in the crystal structure and the short distance between thiol groups corroborated the suitability of this disulfide [28]. Furthermore, refolding experiments using cysteine mutants and diverse redox conditions disclosed the stabilizing effect of the C35^R^–C61^R^ bond on MAT I and MAT III isoenzymes [192].

NEM modification of just two cysteines per subunit inhibited purified rat liver MAT I/III, and this loss of activity correlated with MAT I’s dissociation into inactive MAT III dimers [184,193]. GSSG also inhibited the purified proteins but, in this case, monomers that did not incorporate ^35^S-glutathione were obtained [187], and the data suggested the production of intrasubunit disulfide. Recombinant rat MAT I/III mutants were also used to examine the role of their 10 cysteine residues on activity and association state. Decreased AdoMet synthesis was displayed by C57S, C69S, C105S, and C121S proteins, whereas substitutions on cysteines between C35^R^ and C105^R^ altered the dimer/tetramer ratio [194]. Remarkably, C69 substitution exerted the larger impact on both parameters. It is noteworthy that the five cysteines whose substitution impacts oligomerization belong to the central domain of MATα1, which establishes the contacts that maintain the tetramer [28]; among them, C35^R^ and C61^R^ form the intrasubunit disulfide that blocks MAT I and MAT III interconversion [192]. As C61^R^ is specific to MATα1 (Figure 3), this characteristic may be advantageous for the liver during nutritional overload or stress, allowing the coexistence of isoenzymes with diverse methionine affinities and V_max_.

Similarly, recombinant CBS exhibited lower activity in its oxidized than its reduced forms and both were interconverted by the addition of reducing agents or oxidants. Two (C272 and C275) out of the eleven cysteines of CBS formed a disulfide bond in a CXXC motif of its central domain, both in the recombinant protein and in cells [195]. Mutation of these residues did not preclude heme binding nor change its extent compared with wild-type CBS [195]. Nevertheless, no information on the B_6_ binding was provided, despite the location of the cofactor’s binding site in this central domain. Importantly, exposure of cells to DTT increased their reduced CBS content, in turn resulting in enhanced production of H_2_S [195]. Hence, CBS activity is regulated both by oxidative and reductive stress.

CTH also contains 10 cysteines per subunit. Four of these are included in two conserved CXXC motifs that, according to the homotetramer crystal structure, are relatively exposed at the protein surface (^307^CXXC^310^) and buried close to the dimer–dimer interface (^252^CXXC^255^), respectively. Purified CTH contained the C252–C255 disulfide bond, and this oxidized form showed enhanced activity compared with reduced CTH [189]. This effect could be ascribed to increased cysteine affinity due to a conformational change of the active site, according to results of molecular dynamic simulations and molecular docking. The roles of C252 and C255 in CTH activity were corroborated using mutants, as well as the crucial function of C255 in oxidation sensing. In fact, production of the intramolecular disulfide under oxidative stress required conversion of sulfenylated C255 into a sulfenic acid intermediate that reacted with C252 [189]. As already mentioned, C252 is also targeted by methylglyoxal modification (Figure 6), thus allowing crosstalk between oxidative events leading to disulfide production and this PTM.

### 7.2. Glutathionylation

In spite of inhibition of MAT I/III activity by GSSG, no glutathionylation of the isoenzymes was involved [187]. Conversely, in vitro and in cells, CBS glutathionylation on C346 was found to lead to increased activity [196]. Under the oxidative stress induced by H_2_O_2_ in cells, these effects were transient and precluded in the C346S mutant [196]. It was proposed that this modification favors cysteine production for replenishment of GSH, a decrease in which can be induced by oxidative stress. Interestingly, glutathionylation was identified in CBS monomers and dimers in vitro, but only in monomers of cells under oxidative stress. Additionally, differences in modification efficiency in the presence of GSH or GSSG were detected, suggesting C346 oxidation as a prerequisite for glutathionylation [196]. In vitro CBS glutathionylation prevented further activation by AdoMet, an effect that could have resulted from the presence of C346 in the enzyme’s catalytic domain. Moreover, the crystal structure shows this residue near the dimer interface at the linker that stabilizes the AdoMet-bound conformation of the C-terminal domain.

### 7.3. Nitration and Sulfhydration

MAT I/III, CBS, and CTH are also targets of peroxynitrite, which leads to their inhibition. Inactivation of purified recombinant MAT I/III was prevented in C121S mutant protein but was not precluded by substitution of the acidic and basic residues required for C121 S-nitrosylation [186]. In contrast, CBS inactivation by peroxynitrite in vitro concurred with its nitration on W208, W43, and Y223, leading to changes in heme coordination without alterations of B_6_ binding [197]. Furthermore, results from experiments in the presence of different agents and scavengers suggest that this CBS inhibition may arise partially from decomposition of peroxynitrite into nitrogen dioxide and/or carbonate radicals [197]. Importantly, peroxynitrite is a product of NO and a superoxide radical, and the latter can be generated during Hcy auto-oxidation.

Rat models of diet-induced HHcy and aging have been used to reproduce the increased nitrotyrosine (nitrative stress) and Hcy serum levels often detected during human aging, which are further elevated in subjects with HHcy [198]. These rises correlated with decreased hepatic activity and increased nitration of CBS in the rat models and were improved by pretreatment with a peroxinitrite scavenger [198]. Further analysis of the effects of Hcy in cells expressing diverse CBS protein forms showed decreased activity in only the wild type, and no effect in four tyrosine mutants (Y163A, Y223A, Y381A, and Y518A) [198]. Therefore, a role was suggested for their nitration and in the loss of CBS activity induced by Hcy. Enhanced CBS Tyr nitration was also identified in a mouse model of cerulein-induced pancreatitis [199], but the target residues were not identified. This mouse model presented increased pancreatic *Nos2* expression and protein levels, together with decreased concentrations of several metabolites of sulfur amino acid metabolism (AdoMet, cystathionine, glutathione) and diminished AHCY and CBS protein content compared with the corresponding controls. Moreover, these pancreatitis samples showed no thiol oxidation, but nitration increased due to the AdoMet treatment [199].

HHcy also enhanced CTH nitration levels in animal and cellular models, where it correlated with enzyme inhibition and low H_2_S serum levels [200]. In vitro and in vivo, Hcy-induced nitration was blocked by FeTMPyP, in turn preventing loss of CTH activity, and the role of peroxynitrite was further confirmed in cells treated with an SIN-1 donor. However, the nitrated residues were not reported. That work also described decreased sulfhydration of the Sp1 transcription factor due to Hcy accumulation, leading to alteration in its binding to the *CTH* promoter, subsequently diminishing its transcription [200]. Additionally, CTH sulfhydration was also detected in mouse livers, its level decreasing in HHcy induced by a prolonged high-methionine diet. Using HEK293 cells expressing single CTH mutants, seven residues targeted by sulfhydration (C84, C109, C172, C229, C252, C307, and C310) were identified under control conditions [200]. Additionally, regulation of CTH activity was shown to involve sulfhydration of C84, C109, C229, C252, and C307, while experiments combining agents such as FeTMPyP and/or SIN-1 demonstrated of the need for Hcy-induced nitration to decrease sulfhydration [200]. Other works using mouse liver lysates and a H_2_S donor also documented sulfhydration of CBS, CTH, GNMT, and MATα1 [201], although no further information was provided.

Recently, untargeted and targeted metabolomics of non-small cell lung cancer (NSCLC) cell lines grown in normal or cysteine free media and treated with vehicle or the H_2_S donor GYY4137 revealed important alterations in sulfur amino acid metabolism correlating with ferroptosis, e.g., decreased levels of Hcy [202]. Cells on cystine-free medium also had decreased levels of GSH and cysteine, together with increased amounts of CBS and CTH proteins [202]. Moreover, the addition of GYY4137 further enhanced the CBS protein content. Identification of the sulfhydrated proteins in A549 cells was carried out. Interestingly, AHCY, CBS, GCLC, and GCLM were found among the modified proteins, the latter two being involved in GSH synthesis. In vitro, pull-down and activity assays demonstrated dose-dependent modification and inhibition of recombinant AHCY treated with NaHS [202]. Furthermore, C195 was identified as the main sulfhydrated residue in AHCY, and its role was further confirmed using the C195A mutant. Additional confirmation was obtained from cells expressing this mutant or with C195S knock-in grown in cystine-free or normal media and treated with the H_2_S donor [202]. Moreover, thermal denaturation showed the lower stability of sulfhydrated AHCY versus the unmodified enzyme [202]. Structural data showed C195 located at AdoHcy’s entrance to the active site, and docking experiments led the authors suggest to that sulfhydration would alter this access, thus precluding activity.

## 8. Hotspots for Posttranslational Modification

**Figure 7:**
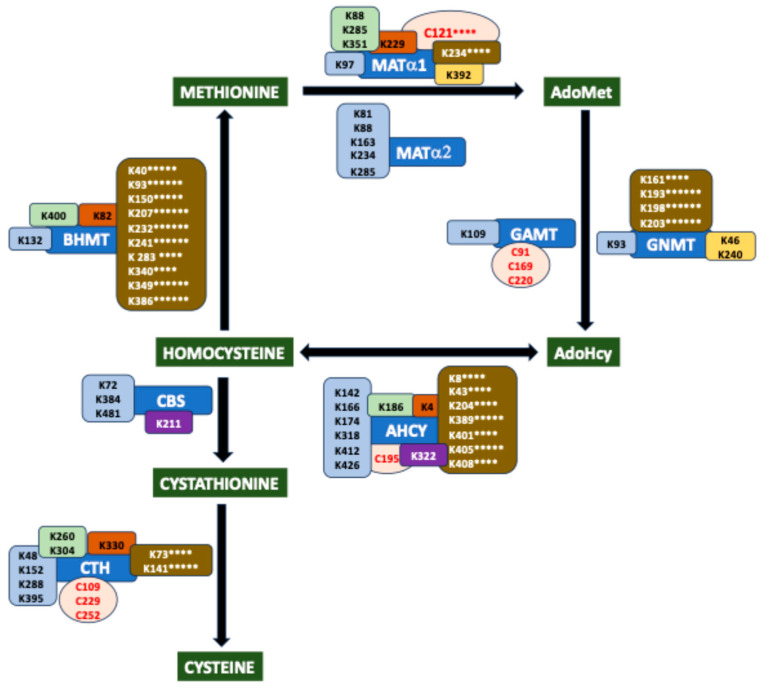
Hotspots for posttranslational modification in enzymes associated with sulfur amino acid metabolism. The figure shows lysine and cysteine residues identified as targets for at least two PTMs in high- and low-throughput studies. The enzymes depicted are only those in which two or more PTMs have been reported on the same residue. Residues of interest are indicated by the following background color code: redox-modified cysteines (salmon); ubiquitylated and acetylated residues (light blue); ubiquitylated, acetylated, and lactylated residues (light green); ubiquitylated, acetylated, and malonylated residues (orange); ubiquitylated, acetylated, and succinylated residues (yellow); ubiquitylated, acetylated, and sumoylated residues (purple); and residues targeted by more than four PTMs (gold), with the number of asterisks denoting how many PTMs have to date been identified at that specific position. Residues targeted by four or more PTMs are indicated by asterisks (*); the number of symbols corresponds to how many modifications have been identified at that position.

The previous sections have described the current knowledge regarding PTMs targeting enzymes of interest, the few cases in which a functional effect has been observed, and the gaps detected in each case. However, no global analysis of the compiled available information has yet been carried out. This section is therefore devoted to providing such a global view, initially highlighting the existence of preferred positions for PTMs (hotspots) in enzymes associated with sulfur amino acid metabolism (Figure 7). The number of such hotspots may appear to be larger for PTMs targeting basic residues than for those involved in redox regulation, although this impression may be biased due to the amount of information currently available. Lysines targeted by six different modifications have so far been identified in GNMT (K193, K198, and K203) and BHMT (K93, K207, K232, K241, K349, and K386), whereas others receiving five PTMs have been reported in AHCY (K389 and K405) and CTH (K141). Such a high incidence of PTMs indicates the key role of that specific position in regulating the behavior of these proteins. Other basic residues in these same enzymes and also in MATα subunits are the targets of four PTMs, while only a small group of ubiquitylation and acetylation sites overlap in GAMT and CBS (Figure 7).

Based on current data, the set of cysteines that are the target of multiple PTMs is limited to C121 in MATα1 and C195 in AHCY. The first of these residues has been identified as the modification site for NO, peroxynitrite, hydroxyl radical, and formaldehyde, all of which inhibit AdoMet synthesis [108,182,183,186]. Meanwhile, AHCY C195 is the modification site for methylglyoxal, formaldehyde, and H_2_S [67,108,202], and its sulfhydration causes inhibition of Hcy production. Furthermore, overlap between cysteines modified by methylgloxal and formaldehyde has been found in GAMT (C91, C169, and C220), whereas similar coincidence has been detected in CTH for methylglyoxal and sulfhydration (C109 and C229) and for methylglyoxal and oxidation (C252). Regulation of CTH activity was shown to depend on these three cysteines [67], whereas no functional effects on AHCY or GAMT have been reported for methylglyoxal or formaldehyde. Nevertheless, although C195 does not seem particularly accessible in the AHCY structure, recent docking data suggested its location in a pocket at the entrance to the active site.

Additionally, no such hotspots can be deduced from the current data on phosphorylation sites, since kinases targeting each site have not been identified. Hence, although several phosphosites have been consistently detected in a variety of samples (Figure 2), it remains unknown whether their modification is the outcome of the action of a single or several kinases.

## 9. Influence of Posttranslational Modification in Pathological States

Putative clinical consequences of PTMs that alter enzyme activity can be envisioned considering the importance of maintaining the AdoMet/AdoHy ratio for transmethylation reactions and preserving the supply of cysteine for GSH synthesis, and also with regard to the need to control Hcy levels and the production of H_2_S. In alcohol-related liver disease, early studies showed reduced AdoMet levels correlating with low MATα1 protein, along with inactivation and dissociation of tetramers in the hepatic cytosol (reviewed in [7,9]). This work is now complemented with data for S144 phosphorylation by casein kinase 2 and K48 sumoylation, modifications that preclude MATα1 mitochondrial translocation and decrease its stability in the cytosol through the MATα1–PIN1 interaction, which is favored by their concurrent action [127]. Moreover, mitochondrial exclusion should also impact the interaction of MATα1 with metabolic proteins in that compartment (e.g., from the TCA cycle), whose outcomes remain unknown. It is noteworthy that neither S144 nor K48 can be considered a MATα1 hotspot based on current data. Remarkably, the effects of either modification on AdoMet synthesis, MATα1 oligomerization state, or nuclear localization, as well as those of PTMs on other enzymes of sulfur amino acid metabolism have not been examined and may exert a significant role in the context of alcohol intoxication.

Data regarding different types of tumors and cancer cell lines have enabled the rendering of a map of PTMs for each enzyme of interest, although their pathological implications remain largely unknown. In HCC and mouse cholangiocarcinoma, MATα1 phosphorylation on S180 and T202 leads to its nuclear accumulation by interfering with YWHAZ interaction [128]. In this compartment, MATα1 interaction with c-Myc regulates its own transcription, which is decreased in HCC. Conversely, MATα2 acetylation on K81 has also been detected in liver carcinoma, promoting ubiquitylation and degradation of this catalytic subunit. Additionally, sumoylated MATα2 collaborates with Bcl-2, stabilizing their mutual interaction and activating the *Bcl2* promoter to regulate apoptosis [162]. Remarkably, this sumoylation occurs on lysines equivalent to those in the MATα1 nucleocytoplasmic signal and the modification levels are higher in nuclear than in cytoplasmic MATα2. Altogether, the simultaneous contributions of these PTMs seem to favor nuclear localization of MAT catalytic subunits, putatively to support epigenetic methylation and/or to collaborate in complexes with transcription factors (e.g., c-Myc or MafK). These effects, along with the well-known *MAT1A*/*MAT2A* expression switch, induce the global AdoMet decrease that occurs in HCC, providing protection from apoptosis. In HCC, the increased production of lactic acid in cancer cells favors lactylation, which takes place in several proteins of sulfur amino acid metabolism and ADK2. However, despite the apparent global regulation of the pathway by this PTM, only the inhibition of adenosine recycling by lactylated ADK2 has been reported, favoring proliferation and metastasis [106]. Other PTMs have also been found in cancer cells, such as CTH ubiquitylation by Rad18 and more extensive methylglyoxal modification, but whether their occurrence is general or specific to certain tumors remains to be evaluated.

Endothelial cells under hypoxia, ligation of mouse carotid arteries, and human artheriosclerotic plaque all induce CTH phosphorylation, with diverse outcomes depending on the modified residue. Hypoxia-related S346 modification of CTH enhanced H_2_S production [145], whereas in the other models, decreased gasotransmitter synthesis arose from S377 phosphorylation by PKG, compromising protection against endothelial dysfunction [149]. Remarkably, the S377 phosphosite is also a key point for O_2_ sensing in the carotid body, allowing crosstalk with NO and CO through guanylate cyclase. Outcomes of decreased H_2_S synthesis in the vasculature also involve CTH ubiquitylation and degradation, which are induced by angiotensin II and contribute to hypertension. These effects are counteracted by K73 acetylation, residue that is targeted by both PTMs. Additionally, conditions such as HHcy, considered a risk factor for cardiovascular disease, decrease serum H_2_S levels in parallel with enhanced CTH nitration and inhibition [200].

Drug intoxication or exposure to a variety of agents that involve GSH dependent detoxification can cause oxidative stress, leading to the variety of the oxidative modifications described in previous sections of this review and also to changes in the subcellular distribution of most enzymes of interest [34]. The pathological consequences of oxidative PTMs may be wider than those of other modifications, since oxidative stress is commonly detected in many diseases affecting major systems. However, the available information mainly concerns decreased AdoMet (e.g., MAT I/III nitrosylation) and H_2_S synthesis (e.g., CBS disulfide bond), while it remains unexplored which PTMs alter subcellular distribution. Altogether, the available information shows many gaps in the knowledge of the regulation by PTM of sulfur amino acid metabolism.

## 10. Concluding Remarks and Future Perspectives

The sophistication of HTP techniques, together with their enhanced sensitivity, allows the massive identification of PTM sites in thousands of proteins. However, these techniques do not enable deciphering the functional consequences of these PTMs on a specific site, and more focused studies may fill these gaps. Additionally, there is limited overlap between identifications obtained from tissues and cell lines, mainly acquired from HEK293 and carcinoma cells. Focusing on information concerning sulfur amino acid metabolism, HTP reports have identified many PTM sites on the main enzymes of the methionine cycle and those related to transsulfuration, although the numbers of residues targeted by a specific modification may vary greatly among enzymes. The existing overlap between residues targeted by diverse PTMs seems more significant among lysines (Figure 5) than cysteines (Figure 6), although this impression may derive from significant differences in the studies carried out to date. The existence of such hotspots highlights the crucial role of specific residues in controlling the targeted enzymes and suggests putative crosstalk between several PTMs acting in the same position. In general, functional information regarding the impact of PTMs in sulfur amino acid metabolism enzymes is very much restricted to a few phosphorylation, acetylation, and redox modification sites that affect activity, and/or the association state, and/or subcellular localization.

Future studies need to focus not only on the identification of the modification sites but also on the functional impact of specific PTMs on enzymes of this pathway in normal and pathological tissues and/or corresponding animal and cellular models. Deciphering the real importance of each PTM site needs to include evaluation of many additional aspects beyond the specific residue that is modified in a certain context. For oligomeric proteins with diverse subcellular localizations and a variety of interaction partners, such as those of sulfur amino acid metabolism, these features should include analysis of the impact of PTMs on the association state, activity, substrate affinities, and changes in subcellular localization and/or protein–protein interactions, as well as the impact of these interactions on the partners involved. Therefore, seeking to gain an in-depth understanding of the contributions of this pathway to health and disease, as well as to find the means to correct its pathological dysfunction, great research efforts should be made in addition to valuable HTP studies.

## Figures and Tables

**Table 1:** Summary of high-throughput studies carried out in cell lines for the identification of posttranslational modifications that include information of enzymes of the mammalian sulfur amino acid metabolism.

PTM	Digestion	Separation/Enrichment	Sample [Ref]
Ubiquitylation ^1^	Lys-C/trypsin	UBISite antibody ^2^	HepG2 [51], Jurkat [51]
		Anti-Gly-Gly	HEK293 [52], HEK293T [53], MV4-11 [53]
	trypsin	Anti-Gly-Gly	Jurkat [54], Jurkat E6-1 [55], HEK293T [56], HCT116 [56], HCT116 [57], HeLa [57], U2OS [58]
Sumoylation	WaLP ^3^	Anti-Gly-Gly	HeLa [57], HCT116 [57]
Acetylation	Lys-C/trypsin	UBISite antibody	HepG2 [51], Jurkat ^4^ [51]
		NS ^5^	HeLa [59]
	trypsin	Anti-acetyl-Lys	Jurkat [54], U2OS [60], Jurkat [61], MV4-11 [61], A549 [61]
		SCX	A2780 [62]
Succinylation	Lys-C/trypsin	Anti-succinyl-Lys	HeLa [59]
	trypsin	Anti-succinyl-Lys	MEFs [63]
Monomethylation	Lys-C/trypsin	Anti-me1Arg ^6^	HEK293T [64]
		PTMScan me1Arg motif	HEK293 [65]
		Anti-me1Lys	KYSE-150 [66]
Methylglyoxal	trypsin	Streptavidin beads	HEK293 [67], HCT116 [67], HeLa [67]
Phosphorylation	trypsin	TiO_2_-beads	U2OS [60], HEK293 [52], HEK293T [68], HeLa [69], HeLa S3 [70], B-cell NHL (11 lines) [71], WM239A [72], A549 [73]
		Fe-IMAC or TiO_2_-beads	HeLa [74]
		IMAC/Anti-phosphoTyr	HeLa S3 [75], GP293 ^11^ [76]
		Fe-IMAC	Jurkat [54], HCT116 [77], SW480 [77], SW620 [77], hESC [78], NIE-115 ^12^ neurites [79]
		IMAC	Jurkat [80], PC9 [81], PC9/gef ^10^ [81], RAW264.7 ^12^ [82]
		Anti-phosphoTyr	Jurkat [83], MV(4;11) [84], Nomo-1 [84], KY821 [84], Molm 14 [84], hESC [85], RAW264.7 ^12^ [86], MEFs ^8^ [87]
		Anti-phosphoSQ/ anti-phosphoTQ	HEK293T [88]
		Anti-CK ^7^ motif	NIH3T3 [89]
		SCX	HL-1 [90]
	Lys-N	SCX	HEK293 [91]
	Lys-C/trypsin	IMAC	HeLa [92], NSCLC ^8^ (16 lines) [93], breast cancer (six lines) [93]
		TiO_2_-beads	HeLa [94], HeLa S3 [95], 3T3-L1 adipocytes [96,97], K562 [94], hESC [98] ^13^
	Glu-C	Anti-11OB7 ^9^	MKN-45 [99]
	Chymotrypsin	Anti-phosphoTyr	MEFs ^9^ [87]

^1^ Trypsinization renders an identical di-glycine remnant for ubiquitin, NEDD8, and ISG15 modifications that are often considered together as ubiquitylations. ^2^ The UBISite monoclonal antibody was generated against the peptide left after Lys-C proteolysis of ubiquitination sites with sequence ESTLHLVLRLRGG. ^3^ WaLP protease cleaves after threonine and rarely after arginine, generating SUMO remnant peptides containing KGG. ^4^ This ubiquitylation study included identification of N-terminal acetylation. ^5^ NS, not specified in Weinert et al. [59]. ^6^ Me1, monomethyl. ^7^ CK, casein kinase. ^8^ MEFs, mouse embryo fibroblasts; NHL, non-Hodgkin lymphoma; NSCLC, non-small cell lung carcinoma. ^9^ Recognizes RxRxxS*/T*, as well as RxxS*/T* motifs. ^10^ Gefinitib-resistant PC9 cells. ^11^ Transfected cells expressing nucleophosmin-ALK wild-type or a catalytic mutant (Y338F/Y342F/Y343F). ^12^ Mouse cell line. ^13^ hESC cells HUES9 and ODENSE-3.

**Table 2:** Summary of high-throughput studies carried out in tissue samples for the identification of posttranslational modifications that include information about enzymes associated with mammalian sulfur amino acid metabolism.

PTM	Digestion	Separation/Enrichment	Sample ^2^ [Ref]
Ubiquitylation ^1^	Lys-C/trypsin	Anti-Gly-Gly	Mouse^BLHKSkM^ [100]
N-glycosylation	Trypsin/Glu-C	Lectins (concanavalin A, wheat germ agglutinin, agglutinin RCA_120_)	Mouse^BLHKPl^ [101]
Acetylation	Lys-C/trypsin	Anti-Acetyl-Lys	Rat^LSPSkMSkThKPfBfBIHLuStTTf^ [102], Human^SkM^ [102]
		NS ^3^	Mouse^L^ [59]
	trypsin	Anti-Acetyl-Lys	Human^L^ [103], Mouse^LC^ [104]
Succinylation	Lys-C/trypsin	Anti-succinyl-Lys	Mouse^L^ [59]
Hydroxybutyrylation	trypsin	Pan-β-hydroxybutyryl-Lys	Mouse^L^ [105]
Lactylation	trypsin	Pan-lactyl-Lys	Human^L^ [106]
Malonylation	trypsin	Anti-malonyl-Lys	Mouse^L^ [107]
Formaldehyde	trypsin		Mouse^L^ [108]
Phosphorylation	Glu-C/trypsin	Ti^4+^-IMAC	Human^L^ [109]
	trypsin	Anti-phosphoTyr	Human cholangiocarcinoma [110]
		Fe-IMAC	Ovarian cancer [111], luminal breast cancer [111], breast cancer xenografts [111], colorectal cancer [77]
		SCX/SAX	Mouse^L^ [112]
		IMAC/Anti-phosphoTyr	Mouse^L^ [113]
		IMAC	Mouse^L^ [114], Mouse^B^ [115], Mouse^BBfHLLuKPST^ [116]
		TiO_2_-beads	Mouse^S^ [117], Rat^L^ [118]
		TiO_2_-beads/Anti-phosphoTyr	Mouse^B^ [119]
	Lys-C/trypsin	Fe^3+^-IMAC	115 Breast tumors [120]
		TiO_2_-beads	Mouse^L^ [121], Rat^PBThHKPfLuSBlTSkMStIL^ [122]
		IMAC	Mouse^L^ mitochondria [123]

^1^ Trypsinization renders an identical di-Gly remnant for ubiquitin, NEDD8, and ISG15 modifications that are often consider together as ubiquitylations. ^2^ Tissues used are indicated by superscripts as follows: B, brain; Bf, brown fat; Bl, blood; Br, breast; C, colon; H, heart; I, intestine; K, kidney; L, liver; Lu, lung; O, ovary; P, pancreas; Pf, perineal fat; Pl, plasma; S, spleen; Sk, skin; SkM, skeletal muscle; St, stomach; T, testis; Tf, testis fat; Th, thymus. ^3^ NS, not specified in Weinert et al. [59].

**Table 3:** Phosphorylation sites identified in enzymes of the mammalian methionine cycle and reverse transsulfuration using high-throughput approaches.

Gene Name	Modification Site ^1,2^ [ref]
*MAT1A*	**S**206 [72], **T**240 [68], **S**247 [68], **S**293 [70], **S**115 [121], **S**115 (mouse^L^) [114]
*MAT2A*	**Y**101 [99], **S**114 [54,69,74,75,96], **S**115 (human^Br^) [120], Y242 [81], **T**262 [95], **S**293 [70], **Y**377 (mouse^L^) [116], **S**384 [54,75,77,78,92,94,111], **S**384 (NSCLC) [93], **S**385 (human^Br^) [120]
*MAT2B*	**S**10 (human^Br^) [120], **Y**175 [98], **Y**186 [98], **S**210 [75], **S**211 (human^Br^) [120], **S**262 [96], **S**263 [96], **T**269 [96], **S**271 [72,78,96], **S**273 (mouse^LK^) [116], **S**273 (mouse) [111], **S**274 (mouse^S^) [116], **T**280 (mouse^P^) [116], **T**280 (NSCLC) [93], **S**282 (mouse^BKP^) [116], **S**282 [54,74,75,77,80,97], **S**282 (mouse^B^) [115,119], **S**282 (mouse^L^) [114], **S**282 (NSCLC, Breast carcinoma) [93], **S**282 (rat^STh^) [122], **S**282 (mouse^S^) [117], **S**282 (human) [111], **S**282 (human^C^) [77], **S**283 (human^Br^) [120], **T**309 [75,94]
*AHCY*	**S**2 [121], **S**3 (human^Br^) [120], **Y**100 [81], **S**154 [54], **T**158 (human^C^) [77], **S**183 [75,77,94,121], **S**183 (mouse^L^) [114], **S**183 (rat^LIKPS^) [122], **S**183 (rat^L^) [118], **T**185 [75], **Y**193 [54,75,76,83,84,85,87], **Y**193 (mouse) [86], **Y**193 (mouse^L^) [113,114,116], **Y**379 [98], **Y**416 [98], **Y**416 (mouse^L^) [113], **S**420 [98], **Y**430 [98]
*GNMT*	**S**10 [121], **S**10 (rat^L^) [118], **S**10 (mouse^L^) [113,114,116], **Y**34 (mouse^L^) [113,114,116], **S**81 [109], **S**163 (mouse^L^) [114], **Y**180 (mouse^L^) [113], **Y**195 (mouse^L^) [113], **Y**196 (human cholangiocarcinoma) [110], **S**197 (mouse^L^) [113], **T**218 (mouse^L^) [113], **Y**221 (mouse^L^)^mit^ [123] ^3^, **Y**221 (mouse^L^) [114], **T**222 (mouse^L^) [113], **T**228 (mouse^L^)^mit^ [123] ^3^, **T**228 (mouse^L^) [116], **S**233 [121], **S**233 (mouse^L^) [114], **S**233 (mouse^LP^) [116], **S**234 (mouse^L^)^mit^ [123] ^3^, **S**235 (human) [111], **S**236 (human^Br^) [120], **Y**243 (mouse^L^) [113], **Y**244 (mouse) [111], **Y**244 (mouse^L^) [114], **T**252 (mouse) [111]
*GAMT*	**S**2 [54], **S**3 (human^Br^) [120], **S**7 (rat^LKI^) [122], **S**18 (human^Br^) [120], **S**30 (mouse^L^)^mit^ [123] ^3^
*MTR*	**S**2 [75], **T**17 [95], **Y**31 [90], **T**136 (human) [111], **S**156 [75], **T**818 [109], **T**834 [79], **S**878 (human^Br^) [120], **Y**1010 [77], **S**1022 [77], **S**1143 (mouse) [111], **S**1182 (mouse) [82], **S**1238 (rat^LHIKLuPSTTh^) [122], **T**1252 [89], **T**1252 (rat^L^) [118], **T**1254 (rat^LHIKLuPSTTh^) [122]
*BHMT*	**T**45 (rat^L^) [118], **T**45 (mouse^L^) [113,114], **Y**77 (mouse^L^)^mit^ [123], **S**79 (mouse^L^)^mit^ [123], **S**79 (mouse^L^) [113,114,116], **S**177 [109], **S**222 (rat^L^) [118], **S**246 (mouse^L^) [114], **S**253 (mouse^L^) [113], **S**330 (mouse^L^)^mit^ [123], **S**330 (mouse^L^) [113,114,116], **S**330 (rat^LBlHIKPT^) [122], **S**330 (rat^L^) [118], **S**333 (mouse^L^)^mit^ [123], **S**333 (mouse^L^) [116], **T**339 (mouse^L^) [114], **Y**363 (mouse^L^) [113], **Y**363 (human cholangiocarcinoma) [110]
*BHMT2*	**S**321 [109], **S**321 (mouse^L^)^mit^ [123], **S**321 (mouse^L^) [113], **S**321 (mouse^LK^) [116], **S**324 (mouse^K^) [116]
*CBS*	**S**3 [75,91], **T**5 [75,91], **T**13 [91], **S**19 [91], **S**23 [91], **S**27 [91,98,109], **S**32 [52,72,95,98], **S**32 (human) [111], **S**33 (human^Br^) [120], **S**42 (rat^LIKLuPTh^) [122], **T**45 (mouse^L^)^mit^ [123], **T**45 (mouse^LKP^) [116], **T**45 (mouse^L^) [114], **S**47 (mouse^L^)^mit^ [123], **S**61 [75], **S**63 [75], **S**120 (mouse^P^) [116], **S**167 [71], **T**181 [71], **T**191 [70,73], **T**192 (human^Br^) [120], **T**193 [70,73], **S**196 (mouse^P^) [116], **S**199 [75,94], **S**200 (human^Br^) [120]
*CTH*	**S**8 [88], **S**16 (rat^L^) [118], **Y**25 (rat^L^) [118], **S**50 (rat^LHIKPSt^) [122], **S**50 (rat^L^) [118], **Y**59 (mouse^L^) [113], **Y**60 [75], **T**105 (rat^L^) [118], **Y**113 (mouse^L^) [113], **S**247 (rat^L^) [118], **S**281 (rat^LHIKPSt^) [122], **S**373 [109], **S**376 [121], **S**376 (mouse^L^) [114], **S**377 (mouse^L^) [112]
*PDRG1*	**S**3 [60,75,95], **S**3 (human^Br^) [120], **S**120 [90], **S**120 (mouse^B^) [119], **S**120 (mouse) [82]

^1^ The type of phosphorylated residue (serine, S; threonine, T; tyrosine, Y) is indicated in bold text. ^2^ The source of mammalian tissue is stated in parentheses. Tissues used are indicated by superscript as follows: B, brain; Bf, brown fat; Bl, blood; Br, breast; C, colon; H, heart; I, intestine; K, kidney; L, liver; Lu, lung; O, ovary; P, pancreas; Pf, perineal fat; Pl, plasma; S, spleen; Sk, skin; SkM, skeletal muscle; St, stomach; T, testis; Tf, testis fat; Th, thymus. ^3^ Analysis carried out in hepatic mitochondrial fraction indicated ‘mit’ in superscript.

**Table 4:** Ubiquitylation sites in enzymes of the mammalian methionine cycle and reverse transsulfuration, identified using high-throughput approaches.

Gene Name	Modification Site ^1^ [ref]
*MAT1A*	**K**48 (mouse^LH^) [100], **K**54 (mouse^LH^) [100], **K**89 (mouse^LH^) [100], **K**98 (mouse^LH^) [100], **K**160 (mouse^L^) [100], **K**222 [51], **K**223 [55], **K**229 (mouse^L^) [100], **K**235 (mouse^LH^) [100], **K**240 [51], **K**266 (mouse^L^) [100], **K**285 [51,55], **K**286 (mouse^L^) [100], **K**289 [52,54,55], **K**303 [51,52,55], **K**304 (mouse^L^) [100], **K**307 [52], **K**308 (mouse^L^) [100], **K**351 [52,53,58], **K**352 (mouse^L^) [100], **K**368 (mouse^L^) [100], **K**369 (mouse^L^) [100], **K**374 (mouse^L^) [100], **K**392 (mouse^L^) [100]
*MAT2A*	**K**18 [51], **K**25 [51], **K**34 [51], **K**39 [51], **K**53 [51], **K**61 [54,55], **K**81 [51,54,55,56,58], **K**81 (mouse^K^) [100], **K**88 [51,54,55,56], **K**97 [51,53,54,55,56], **K**97 (mouse^LKB^) [100], **K**100 [51], **K**102 [51,55], **K**163 [51,54,55], **K**165 [51], **K**171 [51], **K**222 [51], **K**228 [51,54,55,56], **K**228 (mouse^LK^) [100], **K**234 [51,53,54,55,56,57,58], **K**234 (mouse^LKBSkM^) [100], **K**240 [51], **K**244 [51], **K**266 (mouse^L^) [100], **K**285 [51,55], **K**286 (mouse^L^) [100], **K**287 [51], **K**288 [51], **K**289 [52,54,55,56], **K**303 [51,52,55], **K**304 (mouse^L^) [100], **K**307 [51,52,53,54,55], **K**350 [51,53,54,55,56], **K**350 (mouse^LKB^) [100], **K**351 [51,52,53,54,55,57,58], **K**351 (mouse^LKBHSkM^) [100], **K**367 [51,53,54,55], **K**367 (mouse^K^) [100], **K**368 [55], **K**392 [55]
*MAT2B*	**K**35 [51], **K**46 [51,54,55], **K**157 [51], **K**168 [51], **K**180 [51], **K**191 [51], **K**198 [51], **K**209 [51,52,53,54,55,58], **K**209 (mouse^LKB^) [100], **K**214 [51], **K**225 [51,53,54], **K**234 [51], **K**245 [51,53], **K**288 [51], **K**298 [51], **K**299 [51,55], **K**305 [51], **K**315 [51], **K**316 [51,53,54,55], **K**326 [51,53,54,55,56,57,58], **K**326 (mouse^LKB^) [100]
*AHCY*	**K**4 [55], **K**8 [53,55], **K**8 (mouse^L^) [100], **K**15 [51], **K**20 [55,58], **K**20 (mouse^LKBH^) [100], **K**43 [51,52,54,55], **K**43 (mouse^L^) [100], **K**46 [52,54,55], **K**46 (mouse^LKH^) [100], **K**114 [51], **K**138 [51], **K**142 [51], **K**146 [51], **K**158 [51], **K**160 [51], **K**166 [51,53,54,55,56], **K**166 (mouse^LKBHSkM^) [100], **K**174 [51,55,56], **K**174 (mouse^LK^) [100], **K**186 [51,52,54,55,56,57,58], **K**186 (mouse^LKBH^) [100], **K**188 [51,52,53,54,55,56,58], **K**188 (mouse^LKBH^) [100], **K**204 [52,54,55], **K**204 (mouse^LKH^) [100], **K**226 [52,53,54,55,58], **K**226 (mouse^LK^) [100], **K**290 [51], **K**294 [51], **K**318 [51,55], **K**318 (mouse^L^) [100], **K**322 [51,54,55], **K**322 (mouse^L^) [100], **K**331 (mouse^LKH^) [100], **K**361 [51], **K**373 [51], **K**377 [51], **K**380 [51], **K**384 [51], **K**389 [51,55], **K**389 (mouse^LKH^) [100], **K**401 [51,55], **K**401 (mouse^L^) [100], **K**405 [51,52,54,55,56], **K**405 (mouse^LKB^) [100], **K**408 [51,52], **K**412 [51,53], **K**412 (mouse^LK^) [100], **K**426 (mouse^L^) [100]
*GNMT*	**K**46 (mouse^L^) [100], **K**93 (mouse^L^) [100], **K**159 (mouse^L^) [100], **K**191 (mouse^L^) [100], **K**272 (mouse^L^) [100], **K**196 (mouse^LH^) [100], **K**201 (mouse^LH^) [100], **K**238 (mouse^LH^) [100]
*GAMT*	**K**39 [51], **K**39 [55], **K**39 (mouse^LB^) [100], **K**60 [55], **K**109 [51], **K**197 (mouse^L^) [100], **K**235 [55,56], **K**251 (mouse^L^) [100]
*MTR*	**K**15 [55], **K**16 [51,53,55], **K**28 [51,53,54,55,57], **K**45 [51,54,55], **K**57 [51,55], **K**64 [51,55], **K**127 [51,55], **K**139 [51,52,55], **K**151 [51,52,54,55], **K**199 [56], **K**231 [53,55,57], **K**306 [55], **K**309 [51,55], **K**339 [51,55], **K**385 [55], **K**388 [55], **K**466 [55], **K**475 [51,54,55], **K**484 [51,54,55], **K**526 [55], **K**565 [54,55], **K**578 [56], **K**638 [51], **K**645 [51], **K**658 [57], **K**659 [51,55], **K**681 [51,55], **K**685 [51,53,55], **K**699 [51], **K**700 [55], **K**723 (mouse^L^) [100], **K**727 [51,53,54,55], **K**735 [52,54,55], **K**742 [51,55], **K**753 [51,55], **K**781 [55], **K**789 [53,54,55], **K**814 [52,53,55], **K**817 [51,52,53,54,55], **K**823 [55], **K**865 [51,52,53,54,55], **K**871 [51,54,55], **K**925 [55], **K**952 [51], **K**995 [51,55], **K**1000 [51,52,53,54,55], **K**1008 [51], **K**1024 [51], **K**1025 [55], **K**1071 [55], **K**1127 [51,55], **K**1169 [51,55], **K**1186 [51,55], **K**1224 [55], **K**1226 [55], **K**1232 [55], **K**1235 [51,55], **K**1245 [51,55]
*BHMT*	**K**35 (mouse^L^) [100], **K**40 (mouse^LH^) [100], **K**82 (mouse^L^) [100], **K**93 (mouse^LH^) [100], **K**98 (mouse^LH^) [100], **K**132 (mouse^L^) [100], **K**150 (mouse^L^) [100], **K**207 (mouse^L^) [100], **K**229 (mouse^LH^) [100], **K**232 (mouse^LH^) [100], **K**241 (mouse^LH^) [100], **K**283 (mouse^LKH^) [100], **K**340 (mouse^L^) [100], **K**349 (mouse^L^) [100], **K**377 (mouse^LH^) [100], **K**386 (mouse^LH^) [100], **K**400 (mouse^L^) [100]
*BHMT2*	**K**220 (mouse^L^) [100], **K**283 (mouse^LKH^) [100], **K**318 (mouse^L^) [100]
*CBS*	**K**25 (mouse^L^) [100], **K**30 (mouse^H^) [100], **K**48 [51], **K**69 (mouse^LKH^) [100], **K**72 [51], **K**72 (mouse^L^) [100], **K**75 [51], **K**80 (mouse^L^) [100], **K**82 [51,55], **K**83 [51], **K**98 [51], **K**102 [55], **K**116 (mouse^L^) [100], **K**119 [55], **K**134 (mouse^L^) [100], **K**137 [55], **K**158 (mouse^L^) [100], **K**169 (mouse^L^) [100], **K**174 (mouse^L^) [100], **K**177 [52,55], **K**208 (mouse^L^) [100], **K**211 [51,55], **K**319 (mouse^L^) [100], **K**322 [51,55], **K**322 (mouse^L^) [100], **K**325 [55], **K**381 (mouse^L^) [100], **K**384 [55], **K**386 (mouse^LKH^) [100], **K**391 (mouse^L^) [100], **K**394 [53], **K**395 (mouse^L^) [100], **K**398 [51], **K**401 (mouse^L^) [100], **K**477 (mouse^L^) [100], **K**481 [52,54,55], **K**485 [52,55]
*CTH*	**K**4 [51], **K**47 (mouse^L^) [58], **K**48 [51,55], **K**72 (mouse^LKBH^) [58], **K**73 [51,52,53,55], **K**139 [51,55], **K**140 (mouse^LKH^) [58], **K**141 [51], **K**151 (mouse^LKB^) [58], **K**152 [51,56], **K**259, (mouse^LK^) [58], **K**270 (mouse^LK^) [58], **K**288 [51], **K**303 (mouse^L^) [58], **K**304 [55], **K**325 (mouse^L^) [58], **K**329 (mouse^L^) [58], **K**330 [51], **K**333 [55], **K**395 [51]
*PDRG1*	**K**27 [51,55,56], **K**36 [51,52,55,56], **K**74 [56], **K**108 [51,55], **K**112 [51,54,55], **K**125 [51,54,55], **K**128 [55]

^1^ superscript indicates the tissue where the modification was found. Abbreviations used are as follows: L, liver; H, heart; K, kidney; B, brain; SkM, skeletal muscle.

**Table 6:** Acetylation sites in enzymes of the mammalian methionine cycle and reverse transsulfuration, identified using high-throughput approaches.

Gene Name	Modification Site ^1^ [ref]
*MAT1A*	N-term [51], **K**89 (rat^L^) [102], **K**89 (mouse^L^) [59], **K**98 (mouse^L^) [59], **K**229 (rat^L^) [102], **K**235 (rat^L^) [102], **K**235 (mouse^L^) [59,104], **K**286 (mouse^L^) [59], **K**352 (mouse^L^) [59], **K**353 (rat^L^) [102], **K**393 (rat^L^) [102]
*MAT2A*	N-term [51], **K**88 [54], **K**88 (rat^Th^) [102], **K**163 (rat^K^) [102], **K**234 [54,59], **K**234 (rat^L^) [102], **K**286 (mouse^L^) [59]
*AHCY*	N-term [51], **K**4 [54], **K**4 (rat^LuSkMPSTTh^) [102], **K**4 (mouse^L^) [104], **K**8 (rat^LuBK^) [102], **K**8 (mouse^L^) [59], **K**43 (rat^LKLuTTh^) [102], **K**43 (mouse^L^) [59], **K**142 (mouse^L^) [59], **K**166 (rat^LKLuStTTh^) [102], **K**174 (mouse^L^) [59], **K**186 (mouse^L^) [59], **K**204 (rat^LB^) [102], **K**318 (rat^LK^) [102], **K**322 (rat^Th^) [102], **K**322 (mouse^L^) [59], **K**389 (rat^K^) [102], **K**401 [54,59,60,61], **K**401 (rat^L^) [102], **K**401 (mouse^L^) [59], **K**405 (rat^K^) [102], **K**408 [54,59,60,61], **K**408 (rat^LBKLuPSStTh^) [102], **K**408 (human^SkM^) [102], **K**412 (rat^Lu^) [102], **K**412 (mouse^L^) [59], **K**426 (mouse^L^) [59]
*GNMT*	**K**46 (mouse^L^) [59], **K**93 (rat^L^) [102], **K**159rat^L^) [102], **K**159 (mouse^L^) [59], **K**191 (mouse^L^) [104], **K**196 (rat^L^) [102], **K**201 (rat^K^) [102], **K**201 (mouse^L^) [59,104], **K**238 (rat^Sk^) [102], **K**238 (mouse^L^) [59]
*GAMT*	N-term [51], **K**105 (rat^K^) [102], **K**109 (rat^T^) [102]
*MTR*	N-term [51]
*BHMT*	**K**7 (rat^L^) [102], **K**40 (rat^L^) [102], **K**82 (rat^L^) [102], **K**82 (mouse^L^) [59], **K**93 (rat^L^) [102], **K**93 (mouse^L^) [59], **K**132 (mouse^L^) [104], **K**139 (rat^L^) [102], **K**150 (rat^L^) [102], **K**207 (mouse^L^) [104], **K**232 (rat^LK^) [102], **K**232 (mouse^L^) [59,104], **K**241 (mouse^L^) [104], **K**283 (rat^L^) [102], **K**283 (mouse^L^) [59,104], **K**327 (rat^L^) [102], **K**340 (rat^L^) [102], **K**349 (rat^L^) [102], **K**369 (rat^LK^) [102], **K**369 (human^L^) [103], **K**386 (rat^L^) [102], **K**386 (mouse^L^) [59], **K**400 (rat^L^) [102]
*BHMT2*	**K**11 (rat^St^) [102], **K**123 (rat^L^) [102], **K**129 (rat^L^) [102], **K**220 (rat^L^) [102], **K**223 (rat^L^) [102], **K**274 (rat^L^) [102], **K**274 (mouse^L^) [59,104], **K**331 (rat^L^) [102]
*CBS*	**K**72 (rat^BK^) [102], **K**208 (rat^BK^) [102], **K**381 (mouse^L^) [59], **K**386 (rat^K^) [102], **K**386 (mouse^L^) [59], **K**481 [59]
*CTH*	**K**47 (mouse^L^) [59], **K**72 (mouse^L^) [104], **K**140 (mouse^L^) [59,104], **K**140 (rat^LKPTh^) [102], **K**151 (mouse^L^) [59], **K**164 (rat^L^) [102], **K**259 (rat^LP^) [102], **K**287 (rat^LK^) [102], **K**303 (rat^LKPSkSTTh^) [102], **K**329 (rat^LKSkTh^) [102], **K**361 (mouse^L^) [59], **K**383 (rat^LBKT^) [102], **K**394 (rat^KT^) [102]
*PDRG1*	N-term [51]

^1^ Superscripts indicate the tissue where the modification was found, abbreviated as follows: B, brain; K, kidney; L, liver; Lu, lung; P, pancreas; S, spleen; Sk, skin; SkM, skeletal muscle; St, stomach; T, testis; Th, thymus.
